# Vinyl chloride oligomers: On the road to understand the potential toxicity of PVC nanoplastics

**DOI:** 10.1371/journal.pone.0339850

**Published:** 2026-01-12

**Authors:** Esperanza Quevedo, Emiliano Perez-Sanchez, Alexis Caballero, Ana Martínez

**Affiliations:** Departamento de Materiales de Baja Dimensionalidad. Instituto de Investigaciones en Materiales, Universidad Nacional Autónoma de México, Coyoacan, Mexico City, Mexico; VIT University, INDIA

## Abstract

On the road to understand the toxicity of nanoplastics, it is important to determine their capacity to interact with other molecules, as this is the first condition that must be met. In particular, polyvinyl chloride (PVC) is a versatile plastic widely used in construction. It can be degraded producing micro and nanoplastics, which can be formed when PVC pipes are cut during the manufacturing of products. PVC is considered to be one of the most toxic plastics, so it is important to analyze potential detrimental effects. This is the main aim of this research. On the basis of Density Functional Theory calculations, we investigated different vinyl chloride oligomers (as models of PVC nanoplastics). Degradation energies, electron donor acceptor capacities to analyze possible oxidation reactions, and interaction energies with different molecules were calculated. The vinyl chloride oligomers used in this investigation are saturated and monounsaturated. This is important since monounsaturated variant is dominant in experimental conditions. We found that none of the oligomers are good electron donors or acceptors. We also investigated different oligomers interacting with ciprofloxacin and •OOH. The interaction energies with ciprofloxacin and •OOH are negative or less than 13 kcal/mol, indicating weak interactions. This theoretical investigation indicates that vinyl chloride oligomers are not expected to be reactive or toxic, considering the electron transfer and the interaction energies with other molecules.

## Introduction

The polymerization of vinyl chloride produces polyvinyl chloride (PVC), a versatile plastic widely used in construction and to produce pipes, automotive parts, food packaging and traffic lights [1–5]. PVC production is the third largest in the world, representing 16% of total production [6–8]. It is regarded as one of the most toxic plastics [9–13]: In the 1970´s, cancer among workers in the PVC industry and among those living in close proximity to PVC plants, prompted investigations. Results from these studies appeared to indicate that vinyl chloride is a carcinogen and that PVC dust can cause lung cancer. An association between PVC dust and pneumoconiosis was also demonstrated and for this reason, control of PVC dust in industrial settings was prescribed [14–22].

The use of PVC can lead to its fragmentation, producing micro and nanoplastics, which are also formed during the manufacture of products that involve cutting PVC pipes [7,23–25]. PVC micro and nanoplastics have been reported to be frequent contaminants in water and soil [26–29]. This may be dangerous as their potential toxicity is still unknown. Extensive research on the potential toxicity of nanoplastics and on the toxicity of nanoplastics resulting from lung exposure to microplastics revealed potentially dangerous inflammatory responses [21,30–44]. For PVC, reports about potential toxicity are contradictory. As an example, Danso and co-authors [21] conclude that exposure to PVC microplastics has no toxic effects, whereas polystyrene and polypropylene microplastics can cause lung inflammation. Ju and co-authors [30] report that PVC nanoplastics may bind to human serum albumin during the metabolic process and transport *in vitro*, but PVC nanoplastics have not been shown to enter either the digestive or blood circulation systems. If they do, once they enter, nanoplastics might adsorb and bind to serum albumin, possibly affecting protein function and causing serious damage *in vivo*. Recently, an effective methodology for the quantification of nanoplastics in human blood was reported [31], and authors found 1070 ng/ml of polymers in blood. With these results, they verified the presence of nanoplastics in human blood, but due to the small size of nanoplastics and variations in imaging analyses, they concluded that further investigations are needed to fully understand the consequences. In summary, further and in-depth studies are needed to determine the toxicity of PVC nanoplastics *in vivo*.

The reactivity of poly (vinyl chloride) was previously analyzed in the context of chemical recycling [45]. The authors focused on chemical transformations related to preventing deconstruction into monomers and oligomers, which can be used to design recycling processes. They classified the common reaction modes for PVC and noted that C-Cl allylic bonds are more reactive than C-Cl tertiary bonds. PVC contains chlorine atoms that can participate in substitution and elimination reactions, and the authors linked this to the recycling and reuse of PVC. This reactivity could also be related to the potential toxicity of PVC nanoplastics, and this is the main idea of this investigation. Rather than contemplating its recycling processes, we considered the interaction of PVC with molecules to analyze its chemical reactivity and potential toxicity.

Studies that rely on Density Functional Theory (DFT) to understand the free radical polymerization of PVC do exist, but previous investigations did not undertake interaction analysis with other molecules [46]. In our group, we previously investigated, through DFT calculations, the potential toxicity of polyethylene and polyester nanoplastics, and also the potential toxicity of products resulting from PBAT biodegradation [32,33]. We concluded that these nanoplastics may produce oxidative stress and also interact with nitrogen base pairs of DNA. We also reported results for oligomers of polystyrene and polylactic acid [47] and we explain the *Trojan Horse effect*, where pollutants such as antibiotics, can be sequestered by nanoplastics, thereby preventing their oxidation and degradation and promoting bioaccumulation. The interaction of tetracycline with micro and nanoplastics of polyethylene, polystyrene, polypropylene and nylon 6,6 has also been reported [48].

Research on the toxicity of nanoplastics is ongoing, and it is important to analyze the potential toxicity of PVC nanoplastics, which is the main objective of this investigation. The first step is to analyze the electron transfer capacity of vinyl chloride oligomers as models of nanoplastics. We investigated monounsaturated and saturated systems, since it was previously reported that monounsaturated systems are the dominant series remaining after experiments, accounting for 80% of the oligomers [49]. The capacity to accept electrons is important to analyze oxidation reactions as we explain later. Degradation energies were calculated in this investigation in order to analyze the energetic cost of the fragmentation. The second idea is to investigate the interaction of vinyl chloride oligomers with •OOH and ciprofloxacin, to analyze the chemical reactivity of these systems. The chemical reactivity of oligomers is important because one of the conditions for being toxic is being reactive. If they don’t react, they might not be toxic from the perspective of chemical reactivity. Vinyl chloride has been reported as carcinogen, but not the oligomers. PVC dust has also been reported to cause lung cancer, but neither the oligomers nor the nanoplastics have been explicitly labeled as carcinogens. Until now, oligomer analysis is lacking, which is why this research is important. Most PVC plastics will eventually convert to oligomers, so understanding their chemical reactivity is crucial.

## Computational details

To carry out this investigation, we performed Density Functional Theory calculations with the Gaussian16 code [50]. All results were obtained at the theoretical level def2TZVP/ωB97XD [51,52]. This functional accounts for dispersion interactions. Water and acetonitrile were used as solvents to mimic polar and nonpolar environments [53]. Unrestricted calculations were performed and local minima were identified by the number of imaginary frequencies (NIMAG = 0). Several initial conformations were considered, with different approximations of molecules to oligomers forming different bonds, and only the most stable structures are hereby presented. In order to model nanoplastics, we used monounsaturated and saturated vinyl chloride oligomers ranging from two (monomer) up to 14 carbon atoms. Oligomers have been used successfully on previous occasions to analyze various nanoplastics properties [32,33,49,54]. Solvent effects were included during the optimization and in all calculations, using the integral equation formalism of polarizable continuum model (IEF-PCM) with water and acetonitrile for polar and nonpolar environments [55–58].

Degradation energies were calculated considering the formation of the monomer (C_2_H_3_Cl). The chemical equation is:


$${\text{C}}_{\text{x}}{\text{H}}_{\text{y}}{\text{Cl}}_{\text{z}}\;\to {\text{C}}_{\text{2}}{\text{H}}_{\text{3}}\text{Cl}+\;{\text{C}}_{\text{x}-\text{2}}{\text{H}}_{\text{y}-\text{3}}{\text{Cl}}_{\text{z}-\text{1}}$$

and the degradation energies (E_degra_) are calculated as follows:


$${\text{E}}_{\text{degra}}=\;\left[\text{E}\left({\text{C}}_{\text{2}}{\text{H}}_{\text{3}}\text{Cl}\right)+{\text{E}(\text{C}}_{\text{x}-\text{2}}{\text{H}}_{\text{y}-\text{3}}{\text{Cl}}_{\text{z}-\text{1}})\right]-\text{E}({\text{C}}_{\text{x}}{\text{H}}_{\text{y}}{\text{Cl}}_{\text{z}})$$ (1)

To analyze the electron transfer reactions, vertical ionization energy (IE) and vertical electron affinity (EA) are obtained as follows:


$$\text{Y}\;\to {\text{Y}}^{+\text{1}}\;+\;{\text{1e}}^{-}\;\;\;\;\;\;\text{IE}\;=\;\text{E}\;({\text{Y}}^{+\text{1}})\;–\;\text{E}\;(\text{Y})$$ (2)

$${\text{Y}}^{-\text{1}}\;\to \;\text{Y}\;+\;{\text{1e}}^{-}\;\;\;\;\;\;\text{EA}\;=\;\text{E}\;(\text{Y})\;–\;\text{E}\;({\text{Y}}^{-\text{1}})$$ (3)

**Fig 1:**
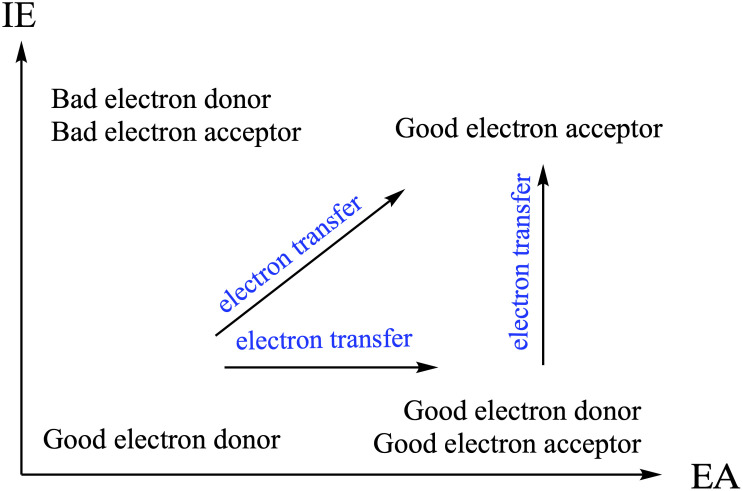
Full Electron Donor-Acceptor Map (FEDAM).

With these values we obtain the Full Electron Donor Acceptor Map (FEDAM) as shown in Fig 1. EA values are plotted in the *X-*axis and IE values are plotted in the *Y-*axis. FEDAM was previously defined and it is a powerful tool to compare the capacity to donate or accept electrons of different systems [59]. Good electron acceptors will have larger ΕΑ values, and good electron donors will present smaller ΙΕ values. We can classify systems using this map, since all molecules with large EA are good electron acceptors (they are on the right side of the map) and those with small IE are good electron donors (in the lower section of the map). All values in the FEDAM are relative. Electrons will be transfer from good electron donors to good electron acceptors, as indicate with the arrows in Fig 1.

An energy index that measures the full electron transfer process [ΔE_FET_] was previously reported [59]. It was derived from chemical reactivity theory and correlates well with the adiabatic Gibbs free energies for the electron transfer processes. ΔE_FET_ is calculated considering the electronegativity (χ) difference plus the arithmetic mean of the hardness (η) values, for electron donators (d) and electron acceptors (a). The equation to calculate ΔE_FET_ is as follows:


$${\Delta \text{E}}_{\text{FET}}=\chi_{\text{d}}-\chi_{\text{a}}+\text{1}/\text{2}(\eta_{\text{d}}+\eta_{\text{a}})$$ (4)

χ_d_ and χ_a_ are the electronegativities of the electron donator and acceptor, respectively;η_d_ and η_a_ are the hardness values of the electron donator and acceptor, respectively. The electronegativities and the hardness are calculated as follows:


χ=½(IE+EA) (5)

η=IE−EA (6)

Interaction energies were obtained by applying the following chemical equation:


XA → X + A

X are the oligomers and A are molecules, and the interaction energy (E_int_) is calculated as follows:


$${\text{E}}_{\text{int}}\;=\;[\text{E}(\text{X})+\text{E}(\text{A})–\;\text{E}(\text{AX})$$ (7)

Positive values indicate that XA is more stable than A + X, and therefore the interaction is energetically viable.

## Results and discussion

**Fig 2:**
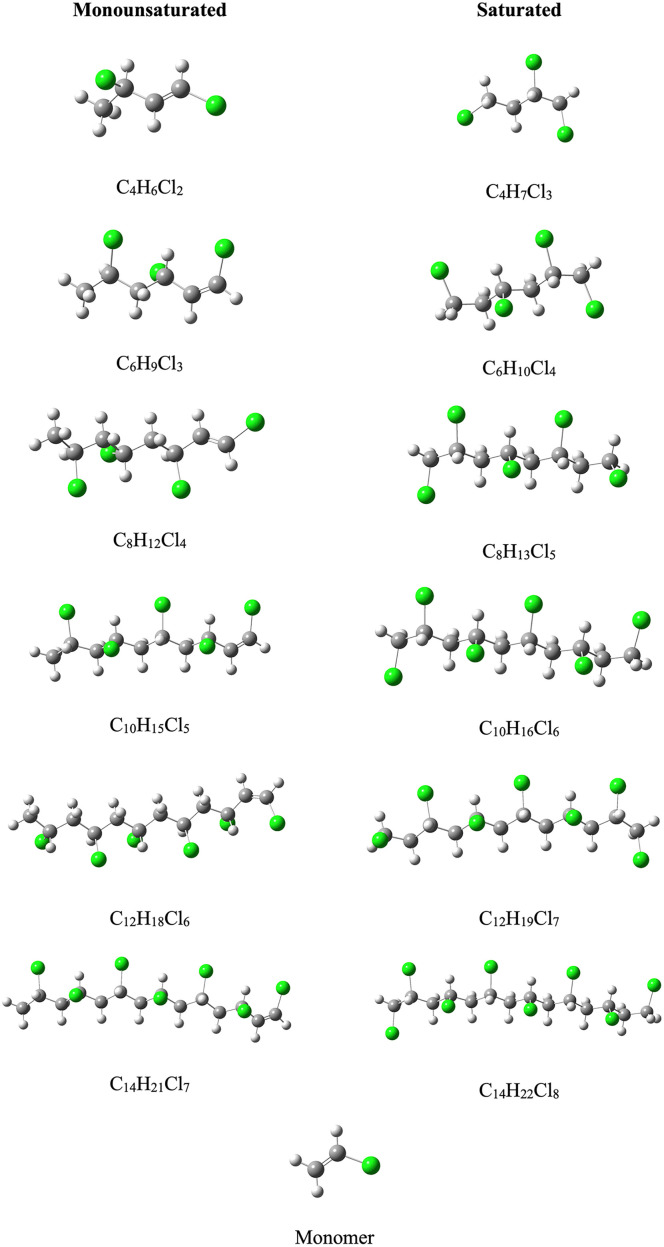
Optimized structures (in water) of the systems under study. Carbon atoms in gray color, chlorine atoms in green and hydrogen atoms in white. Molecular formulas are included to aid identification.

The optimized structures of all the oligomers that we analyzed are presented in Fig 2. According to the experimental information, oligomers present linear structures that comply with those obtained theoretically [45]. The chlorine atoms are arranged in staggered order to avoid steric effects. The length of the oligomers ranges from 0.60 to 1.80 nm. In all oligomers, the C-C bond distance is 1.5 Å and the C-Cl bond distance is equal to 1.8 Å. In these optimized structures, chlorine atoms are separated by more than 4 Å. We considered saturated and monounsaturated oligomers. The double C-C bond is located between C1 and C2. We considered other positions of the double bond but the structures are less stable. The optimized structures reported in Fig 2 are obtained in water. Those optimized in acetonitrile are quite similar and are reported in the S1 Table.

**Fig 3:**
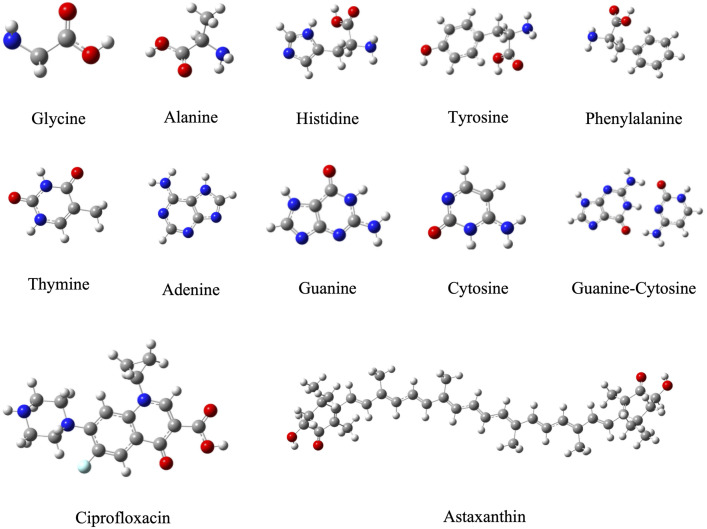
Optimized structures (in water) of the molecules and biomolecules under study. Carbon atoms in gray color, oxygen atoms in red, nitrogen atoms in blue, fluorine atom in light blue and hydrogen atoms in white.

In Fig 3 the optimized structure of molecules and biomolecules are reported. We investigate five amino acids [glycine (Gly), alanine (Ala), histidine (His), tyrosine (Tyr) and phenylalanine (Phenyl)]; four nitrogen bases [thymine (T), adenine (A), guanine (G) and cytosine (C)]; the guanine-cytosine base pair (GC); one antibiotic (ciprofloxacin); and one antioxidant [astaxanthin (ASTA)]. These amino acids were chosen as they have different functional groups. GC was selected as it has the highest intermolecular binding energy of all base pairs [60]. Ciprofloxacin is a common antibiotic which is recommended by the World Health Organization’s list of essential medicines [61], and experimental studies have shown the interactions between various nanoplastics and ciprofloxacin [62,63]. ASTA is a well-established antioxidant with wide commercial use [64]. The optimized structures in acetonitrile are similar and are also included in the S1 Table.

Table 1 shows the degradation energies of vinyl chloride oligomers. All values are close to 30 kcal/mol, meaning that the binding energy of a monomer is similar in all cases, regardless of the size of the oligomer’s backbone. Analyzing the values, 30 kcal/mol is not a large amount of energy in a thermochemical or bioenergetic context. The energy released in the oxidation of glucose is 686 kcal/mol. The binding energy of most organic molecules is significantly greater than 30 kcal/mol. A two-meter-high, 14-meter-long ocean wave could contain about 45 kWh of energy (about 38,000 kcal), which is much more than the degradation energy of vinyl chloride oligomers. Therefore, degradation of vinyl chloride oligomers can be expected under normal environmental conditions.

**Table 1:** Degradation energies (E_degra_) of the vinyl chloride oligomers that we investigate. Values are reported in kcal/mol.

C_x_H_y_Cl_z_ → C_2_H_3_Cl + C_x-2_H_y-3_Cl_z-1_		
MONOUNSATURATED	E_degra_ (water)	E_degra_ (acetonitrile)
C_4_H_6_Cl_2_	31.52	28.14
C_6_H_9_Cl_3_	29.51	27.42
C_8_H_12_Cl_4_	28.78	30.09
C_10_H_15_Cl_5_	31.41	28.70
C_12_H_18_Cl_6_	30.03	28.79
C_14_H_21_Cl_7_	30.13	28.14
SATURATED		
C_6_H_10_Cl_4_	30.51	29.15
C_8_H_13_Cl_5_	30.13	28.79
C_10_H_16_Cl_6_	30.16	28.81
C_12_H_19_Cl_7_	30.00	28.67
C_14_H_22_Cl_8_	30.06	28.72

**Fig 4:**
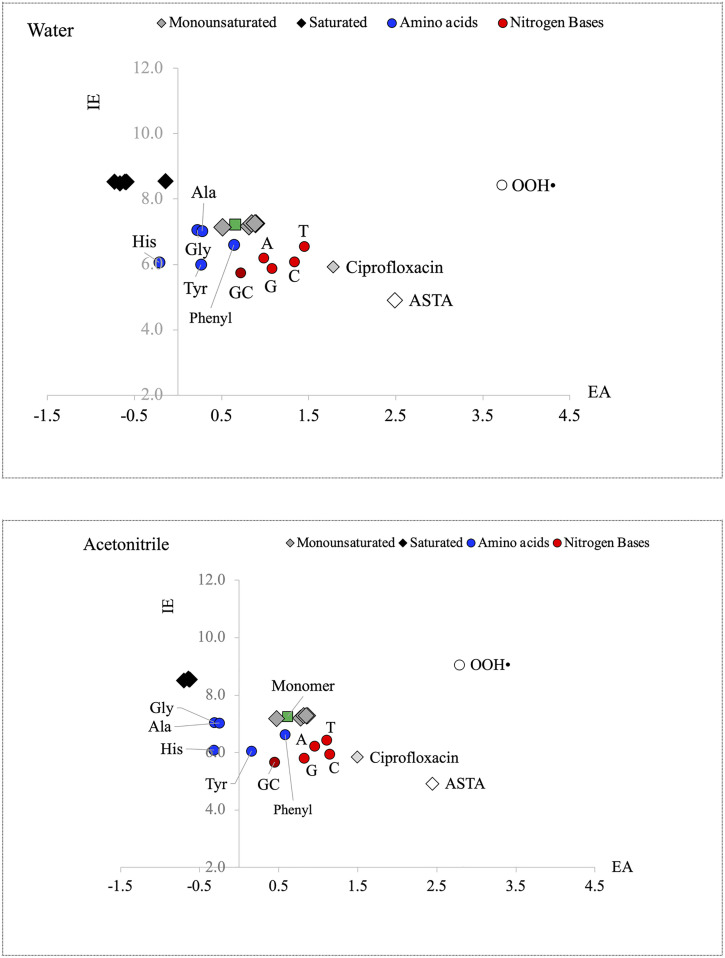
FEDAM for the systems under study. Water and acetonitrile are used to mimic polar and nonpolar environments. All values in eV.

For the analysis of the electron transfer process, in Fig 4 we present the FEDAM for the studied compounds. As can be seen, results in water and acetonitrile are comparable. We also include a well-known free radical (•OOH) closely related to oxidative stress for comparison. The mechanism of action of •OOH is based on the electron transfer process. It is a good electron acceptor that extracts electrons from other molecules, oxidizing them. This is in agreement with the results of the FEDAM. •OOH is the best electron acceptor among all the studied systems, followed by ASTA and ciprofloxacin. As Fig 1 indicates, electron will be transfer from systems that are good electron donors to those systems that are good electron acceptors. All saturated oligomers present negative values of EA, in water and in acetonitrile. This means that they are not good electron acceptors. There are also amino acids (His, Gly and Ala) that present negative values of EA and they are also bad electron acceptors. Unsaturated oligomers are bad electron donors (IE is large) and bad electron acceptors (EA is small), but they are better donors and acceptors than the saturated oligomers. Nitrogen bases are better electron acceptors than amino acids and oligomers, but they are not as good as ASTA, ciprofloxacin or •OOH. The only systems that can be considered good electron acceptors are ASTA and •OOH. They could accept electrons from all the other molecules, however, all other molecules have high IE values and so they are poor electron donors, meaning that any electron transfer is unlikely to occur. With the results reported in Fig 4, it is possible to conclude that unsaturated and saturated oligomers are not good electron donor or acceptors. Values for biomolecules and other molecules are similar to the values of the oligomers, and so the electron transfer process with these vinyl chloride oligomers is difficult to achieve without energy costs. To corroborate this idea, we obtain the energy index that measures the full electron transfer process, according to equation 4. Results are reported in Tables 2 (water) and 3 (acetonitrile). Two scenarios were considered: with vinyl chloride oligomers as electron donators and with vinyl chloride oligomers as electron acceptors. As is shown, all values are positive, and this means that the electron transfer reactions are not spontaneous. Environmental factors such as temperature or atmospheric pressure may promote electron transfer reactions, but considering these variables is beyond the scope of this investigation. Under the conditions of our calculations, electron transfer reactions are not thermodynamically spontaneous (Tables 2 and 3).

**Table 2:** Energy index (ΔE_FET_) that measures the full electron transfer process for all oligomers under study and some molecules analyzed in water. All values in eV.

Water			ΔΕ_FET_ = χ_oli_ – χ_molec_ + 1/2(η_oli_ + η_molec_)								
Monounsaturated	GC	Gly	Ala	Phenyl	Tyr	His	G	C	A	T	•OOH
Monomer	6.51	7.00	6.95	6.58	6.96	7.44	6.15	5.89	6.24	5.77	3.51
C_4_H_6_Cl_2_	6.45	6.94	6.89	6.52	6.90	7.38	6.09	5.82	6.18	5.71	3.45
C_6_H_9_Cl_3_	6.52	7.02	6.97	6.60	6.98	7.46	6.16	5.90	6.26	5.79	3.53
C_8_H_12_Cl_4_	6.42	6.92	6.87	6.50	6.88	7.36	6.06	5.80	6.16	5.69	3.43
C_10_H_15_Cl_5_	6.53	7.03	6.97	6.60	6.98	7.46	6.17	5.91	6.26	5.79	3.53
C_12_H_18_Cl_6_	6.54	7.04	6.98	6.61	6.99	7.47	6.18	5.92	6.27	5.80	3.54
C_14_H_21_Cl_7_	6.54	7.03	6.98	6.61	6.99	7.47	6.17	5.91	6.27	5.80	3.54
Saturated	GC	Gly	Ala	Phenyl	Tyr	His	G	C	A	T	•OOH
C_4_H_7_Cl_3_	7.83	8.33	8.27	7.91	8.29	8.76	7.47	7.21	7.56	7.10	4.83
C_6_H_10_Cl_4_	7.75	8.25	8.20	7.83	8.21	8.69	7.39	7.13	7.49	7.02	4.75
C_8_H_13_Cl_5_	7.81	8.30	8.25	7.88	8.26	8.74	7.45	7.19	7.54	7.07	4.81
C_10_H_16_Cl_6_	7.81	8.30	8.25	7.88	8.26	8.74	7.45	7.19	7.54	7.07	4.81
C_12_H_19_Cl_7_	7.81	8.30	8.25	7.88	8.26	8.74	7.45	7.19	7.54	7.07	4.81
C_14_H_22_Cl_8_	7.80	8.30	8.24	7.88	8.25	8.73	7.44	7.18	7.53	7.07	4.80
**Water**			**ΔΕ_FET_ = χ_molec_ – χ_oli_ + 1/2(η_oli_ + η_molec_)**								
Monounsaturated	GC	Gly	Ala	Phenyl	Tyr	His	G	C	A	T	•OOH
Monomer	5.08	6.40	6.37	5.94	5.34	5.40	5.22	5.42	5.54	5.90	7.77
C_4_H_6_Cl_2_	4.92	6.25	6.21	5.79	5.19	5.25	5.06	5.26	5.39	5.74	7.62
C_6_H_9_Cl_3_	4.84	6.16	6.13	5.71	5.11	5.17	4.98	5.18	5.31	5.66	7.54
C_8_H_12_Cl_4_	5.23	6.55	6.51	6.09	5.49	5.55	5.36	5.57	5.69	6.04	7.92
C_10_H_15_Cl_5_	4.89	6.21	6.18	5.75	5.15	5.21	5.03	5.23	5.35	5.71	7.58
C_12_H_18_Cl_6_	4.84	6.16	6.13	5.70	5.10	5.16	4.97	5.18	5.30	5.66	7.53
C_14_H_21_Cl_7_	4.85	6.17	6.14	5.71	5.11	5.17	4.99	5.19	5.31	5.67	7.54
Saturated	GC	Gly	Ala	Phenyl	Tyr	His	G	C	A	T	•OOH
C_4_H_7_Cl_3_	5.88	7.20	7.17	6.74	6.14	6.20	6.02	6.22	6.34	6.70	8.57
C_6_H_10_Cl_4_	6.40	7.72	7.69	7.26	6.67	6.72	6.54	6.74	6.87	7.22	9.10
C_8_H_13_Cl_5_	6.34	7.66	7.63	7.21	6.61	6.67	6.48	6.68	6.81	7.16	9.04
C_10_H_16_Cl_6_	6.33	7.65	7.62	7.19	6.59	6.65	6.47	6.67	6.79	7.15	9.02
C_12_H_19_Cl_7_	6.46	7.79	7.75	7.33	6.73	6.79	6.60	6.80	6.93	7.28	9.16
C_14_H_22_Cl_8_	6.33	7.65	7.62	7.19	6.60	6.66	6.47	6.67	6.80	7.15	9.03

**Table 3:** Energy index (ΔE_FET_)that measures the full electron transfer process for all oligomers under study and some molecules analyzed in acetonitrile. All values in eV.

Acetonitrile			ΔΕ_FET_ = χ_oli_ –χ_molec_ + 1/2(η_oli_ + η_molec_)								
Monounsaturated	GC	Gly	Ala	Phenyl	Tyr	His	G	C	A	T	•OOH
Monomer	6.82	7.58	7.52	6.69	7.11	7.59	6.44	6.12	6.32	6.16	4.48
C_4_H_6_Cl_2_	6.76	7.52	7.46	6.63	7.05	7.53	6.38	6.06	6.25	6.10	4.42
C_6_H_9_Cl_3_	6.84	7.60	7.54	6.70	7.13	7.61	6.46	6.14	6.33	6.18	4.50
C_8_H_12_Cl_4_	6.74	7.50	7.44	6.61	7.03	7.51	6.36	6.05	6.24	6.08	4.40
C_10_H_15_Cl_5_	6.84	7.60	7.54	6.71	7.13	7.61	6.47	6.15	6.34	6.18	4.51
C_12_H_18_Cl_6_	6.85	7.61	7.55	6.72	7.14	7.62	6.48	6.16	6.35	6.19	4.52
C_14_H_21_Cl_7_	6.85	7.61	7.55	6.72	7.14	7.62	6.47	6.15	6.35	6.19	4.51
Saturated	GC	Gly	Ala	Phenyl	Tyr	His	G	C	A	T	•OOH
C_4_H_7_Cl_3_	7.83	8.33	8.27	7.91	8.29	8.90	7.75	7.44	7.63	7.47	5.79
C_6_H_10_Cl_4_	7.75	8.25	8.20	7.83	8.21	8.83	7.69	7.37	7.56	7.40	5.73
C_8_H_13_Cl_5_	7.81	8.30	8.25	7.88	8.26	8.88	7.73	7.41	7.61	7.45	5.77
C_10_H_16_Cl_6_	7.81	8.30	8.25	7.88	8.26	8.88	7.74	7.42	7.61	7.45	5.78
C_12_H_19_Cl_7_	7.81	8.30	8.25	7.88	8.26	8.88	7.73	7.41	7.61	7.45	5.77
C_14_H_22_Cl_8_	7.80	8.30	8.24	7.88	8.25	8.87	7.73	7.41	7.60	7.44	5.77
**Acetonitrile**			**ΔΕ_FET_ = χ_molec_ – χ_oli_ + 1/2(η_oli_ + η_molec_)**								
Monounsaturated	GC	Gly	Ala	Phenyl	Tyr	His	G	C	A	T	•OOH
Monomer	5.07	6.44	6.42	6.02	5.44	5.48	5.20	5.35	5.61	5.83	8.45
C_4_H_6_Cl_2_	4.90	6.27	6.25	5.85	5.28	5.31	5.04	5.18	5.45	5.66	8.28
C_6_H_9_Cl_3_	4.82	6.19	6.18	5.78	5.20	5.23	4.96	5.10	5.37	5.58	8.21
C_8_H_12_Cl_4_	5.20	6.58	6.56	6.16	5.58	5.62	5.34	5.48	5.75	5.96	8.59
C_10_H_15_Cl_5_	4.86	6.24	6.22	5.82	5.24	5.28	5.00	5.14	5.41	5.62	8.25
C_12_H_18_Cl_6_	4.81	6.18	6.17	5.77	5.19	5.22	4.95	5.09	5.36	5.57	8.20
C_14_H_21_Cl_7_	4.83	6.20	6.18	5.78	5.20	5.24	4.96	5.11	5.37	5.59	8.21
Saturated	GC	Gly	Ala	Phenyl	Tyr	His	G	C	A	T	•OOH
C_4_H_7_Cl_3_	6.32	7.69	7.67	7.27	6.70	6.73	6.46	6.60	6.87	7.08	9.71
C_6_H_10_Cl_4_	6.38	7.75	7.73	7.33	6.75	6.79	6.51	6.66	6.92	7.13	9.76
C_8_H_13_Cl_5_	6.31	7.69	7.67	7.27	6.69	6.73	6.45	6.59	6.86	7.07	9.70
C_10_H_16_Cl_6_	6.30	7.67	7.65	7.25	6.68	6.71	6.44	6.58	6.85	7.06	9.69
C_12_H_19_Cl_7_	6.31	7.68	7.66	7.26	6.68	6.72	6.44	6.59	6.86	7.07	9.69
C_14_H_22_Cl_8_	6.30	7.68	7.66	7.26	6.68	6.72	6.44	6.58	6.85	7.06	9.69

The capacity to accept electrons is related to oxidative stress. Systems accept electrons that others lose. Oxidation is the loss of electrons, so molecules or systems that lose electrons are oxidized. This is one of the mechanisms of oxidative stress. With the results reported here, it can be concluded that vinyl chloride oligomers do not promote oxidative stress through the electron transfer mechanism. This could be due to other mechanisms, which undoubtedly deserve to be analyzed in future research, but not considering their oxidizing capacity.

**Fig 5:**
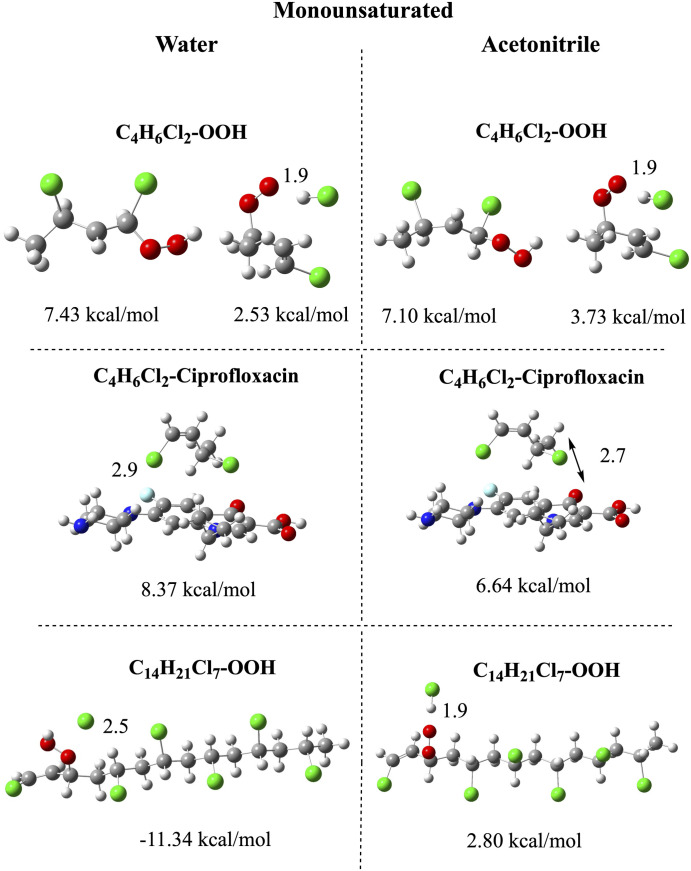
Optimized structures and interaction energies of monounsaturated oligomers (the smallest and the largest) with •OOH. The smallest oligomer is also with ciprofloxacin. Interatomic distances are reported in Å. Water and acetonitrile are used to mimic polar and nonpolar environments. Carbon atoms in gray color, chlorine atoms in green, hydrogen atoms in white, nitrogen atoms in blue, oxygen atoms in red, and fluorine atoms in light blue.

**Fig 6:**
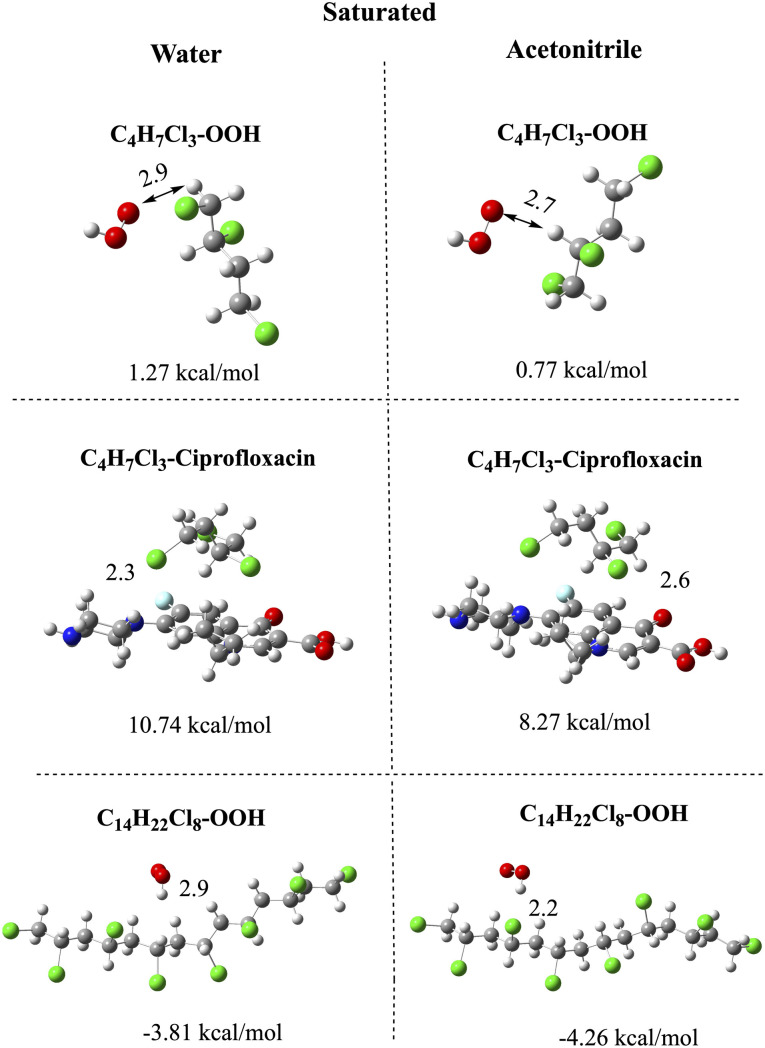
Optimized structures and interaction energies of monounsaturated oligomers (the smallest and the largest) with •OOH. The smallest oligomer is also with ciprofloxacin. Interatomic distances are reported in Å. Water and acetonitrile are used to mimic polar and nonpolar environments. Carbon atoms in gray color, chlorine atoms in green, hydrogen atoms in white, nitrogen atoms in blue, oxygen atoms in red, and fluorine atoms in light blue.

To analyze the chemical reactivity of the oligomers, we analyze the interaction of the smallest oligomers (C_4_H_6_Cl_2_ and C_4_H_7_Cl_3_) with the best electron acceptors according to the FEDAM (•OOH) and with ciprofloxacin. Ciprofloxacin was selected since it coexists with nanoplastics in the environment. We also investigate the largest oligomers (C_14_H_21_Cl_7_ and C_14_H_22_Cl_8_) interacting with •OOH. The idea behind this selection is to see the influence of oligomer size on chemical reactivity. Figs 5 and 6 report the optimized structures of all the systems, the interaction energies and selected bond distances. It is worth noting that other initial geometries were also studied, with the molecules interacting with different sections of the vinyl chloride oligomers, but the resulting optimized structures were less stable than those reported in Figs 5 and 6. All interaction energies are less than 13 kcal/mol or negative, indicating either weak or energetically unfavorable interactions. The optimized structures of the compounds feature different bonds. For monounsaturated oligomers (Fig 5), the interaction with •OOH produces two optimized geometries: one of the structures has the •OOH bonded to the double bond with an interaction energy of 7.43 or 7.10 kcal/mol (in water and acetonitrile, respectively). The other optimized structures present a chlorine atom dissociated to form HCl, with a lower interaction energy (2.53 and 3.73 kcal/mol, in water and acetonitrile, respectively). The dissociation of the chlorine atom is also observed for the largest oligomers interacting with •OOH. For these large oligomers, the structure with the •OOH bonded to the double C-C bond was not found. In water, the interaction energy with C_14_H_21_Cl_7_ is negative, meaning that it is not energetically feasible. In acetonitrile, the interaction energy of the largest oligomer is lower than that of the smallest oligomer. This indicates that the largest oligomer is less reactive than the smaller one. The dissociation of Cl and formation of HCl is not surprising since PVC is well known to be prone to thermal degradation, a process in which it loses hydrogen chloride (HCl). It seems that the interaction of these oligomers with •OOH is similar to thermal degradation in the production of chlorine atoms or HCl. The idea that emerges from these results is that we can analyze the possibilities of functionalizing these nanoplastics through interaction with free radicals, to investigate whether aging or degradation of the plastic can generate functionalized nanoplastics. Until now, there is no evidence about the generation of functionalized nanoplastics after degradation, which could be related to toxicity. This research is ongoing.

In Fig 5 we also report the interaction of ciprofloxacin with the smallest monounsaturated oligomer. The interaction energies are positive in water and acetonitrile (8.37 and 6.64 kcal/mol, respectively) but less than 10 kcal/mol. Therefore, it is not possible to conclude that these oligomers are highly reactive.

Saturated oligomers (Fig 6) interact with molecules forming hydrogen bonds in all cases. The bond distance of the hydrogen bonds is large, showing a weak bond. This is consistent with the interaction energies, all less than 11 kcal/mol and even negative in some cases. The interaction with ciprofloxacin is more favorable than the interaction with •OOH, and in all systems the interaction in water is more favorable than in acetonitrile. With the results of Fig 6, it is not possible to conclude that oligomers are highly reactive. All oligomers analyzed in this investigation (monounsaturated and saturated) have low, even negative, interaction energies, indicating that the interaction is not energetically favorable for any of these two oligomers.

## Conclusions

What is the chemical reactivity of PVC nanoplastics? The results from this theoretical study provide some answers. The electron transfer properties of the oligomers studies here indicate that it is unlikely that vinyl chloride oligomers participate in the oxidative stress processes trough the electron transfer mechanism. These oligomers are neither good electron acceptors nor good electron donors. Vinyl chloride oligomers interact with some molecules, but the interaction energy is smaller than 13 kcal/mol, so it cannot be considered that these oligomers are highly reactive. These results indicate that there is not a potential risk to human and animal health due to the presence of vinyl chloride oligomers that involves either an electron transfer or interaction energies. This theoretical investigation constitutes a good starting point for a more in-depth analysis of the toxicity of PVC nanoplastics.

## Supporting information

S1 Table Cartesian coordinates of all systems under consideration, along with the level of theory associated with each calculation. (PDF)
