# Supplementary material for: Vinyl chloride oligomers: On the road to understand the potential toxicity of PVC nanoplastics
Source: PLoS One. 2026 Jan 12;21(1):e0339850. doi: 10.1371/journal.pone.0339850 (PMC12863054; doi:10.1371/journal.pone.0339850)
Supplement: S1 Table — (PDF) [file pone.0339850.s001.pdf]

S1 Table. Cartesian coordinates of all systems under consideration, along with the level of theory associated with each calculation.

Monomer in water

opt freq wb97xd scrf=(smd,solvent=water) def2tzvp

Standard orientation:

Center Number	Atomic Number	Atomic Type	Coordinates (Angstroms) X	Y	Z
1	6	0	-0.573459	0.503039	-0.000000
2	1	0	-0.618579	1.583400	0.000000
3	6	0	-1.628293	-0.285908	-0.000000
4	1	0	-1.550305	-1.365893	-0.000000
5	1	0	-2.614824	0.160976	0.000001
6	17	0	1.058484	-0.098898	0.000000

Monomer in acetonitrile

opt freq wb97xd scrf=(smd,solvent=acetonitrile) def2tzvp

Standard orientation:

Center Number	Atomic Number	Atomic Type	Coordinates (Angstroms) X	Y	Z
1	6	0	-0.573627	0.502770	-0.000000
2	1	0	-0.618755	1.583444	0.000000
3	6	0	-1.628952	-0.285786	-0.000000
4	1	0	-1.551788	-1.366107	-0.000000
5	1	0	-2.615554	0.161702	0.000001
6	17	0	1.058916	-0.098879	0.000000

$C_4H_6Cl_2$ in water

opt freq wb97xd scrf=(smd,solvent=water) def2tzvp

Standard orientation:

Center Number	Atomic Number	Atomic Type	Coordinates (Angstroms) X	Y	Z

1	6	0	-1.104939	0.403062	0.350086
2	1	0	-1.053193	0.388702	1.436013
3	6	0	-1.799342	1.651694	-0.146993
4	1	0	-1.891580	1.641027	-1.233922
5	1	0	-2.790879	1.746774	0.294754
6	1	0	-1.205661	2.521329	0.142879
7	17	0	-2.122348	-1.059072	-0.075445
8	6	0	0.247592	0.213771	-0.245251
9	1	0	0.323233	0.247001	-1.327081
10	6	0	1.314744	0.042816	0.513053
11	1	0	1.290838	0.003942	1.594073
12	17	0	2.909342	-0.141919	-0.144089

$C_4H_6Cl_2$ in acetonitrile

opt freq wb97xd scrf=(smd,solvent= acetonitrile) def2tzvp

Standard orientation:

Center Number	Atomic Number	Atomic Type	Coordinates (Angstroms) X	Y	Z
1	6	0	-1.105425	0.403052	0.349372
2	1	0	-1.053939	0.389249	1.435571
3	6	0	-1.799915	1.652548	-0.147209
4	1	0	-1.893715	1.642785	-1.234265
5	1	0	-2.791332	1.747939	0.295500
6	1	0	-1.205863	2.522377	0.142505
7	17	0	-2.123621	-1.059461	-0.075058
8	6	0	0.247861	0.213188	-0.245435
9	1	0	0.323629	0.243755	-1.327553
10	6	0	1.315569	0.043838	0.512826
11	1	0	1.292233	0.006501	1.594172
12	17	0	2.910706	-0.142207	-0.143957

$C_6H_9Cl_3$ in water

opt freq wb97xd scrf=(smd,solvent=water) def2tzvp

Standard orientation:

Center Number	Atomic Number	Atomic Type	Coordinates (Angstroms) X	Y	Z

1	6	0	2.186220	-0.097040	-0.156461
2	1	0	2.323271	-0.833094	0.632618
3	6	0	3.400712	-0.052508	-1.054500
4	1	0	3.289014	0.710678	-1.826496
5	1	0	4.305191	0.152178	-0.482715
6	1	0	3.514603	-1.023303	-1.541665
7	17	0	2.043274	1.487194	0.737550
8	6	0	0.910863	-0.372319	-0.928962
9	1	0	1.060390	-1.300091	-1.487830
10	1	0	0.754347	0.423808	-1.662567
11	6	0	-0.361521	-0.484355	-0.108926
12	6	0	-1.552145	-0.723875	-0.968313
13	1	0	-1.466501	-1.507252	-1.714568
14	6	0	-2.697660	-0.066352	-0.925077
15	1	0	-3.521125	-0.277564	-1.591874
16	17	0	-3.050493	1.214078	0.192016
17	17	0	-0.232748	-1.875191	1.070862
18	1	0	-0.498555	0.389974	0.521239

$C_6H_9Cl_3$ in acetonitrile

opt freq wb97xd scrf=(smd,solvent= acetonitrile) def2tzvp

Standard orientation:

Center Number	Atomic Number	Atomic Type	Coordinates (Angstroms) X	Y	Z
1	6	0	2.185819	-0.092951	-0.153909
2	1	0	2.323393	-0.827404	0.636824
3	6	0	3.401502	-0.050038	-1.051452
4	1	0	3.290700	0.711000	-1.826087
5	1	0	4.305497	0.157166	-0.479232
6	1	0	3.517460	-1.022375	-1.535741
7	17	0	2.041913	1.493283	0.737258
8	6	0	0.910222	-0.370899	-0.926383
9	1	0	1.062668	-1.296499	-1.488457
10	1	0	0.750376	0.426885	-1.657755
11	6	0	-0.362131	-0.490563	-0.106021
12	6	0	-1.554118	-0.722422	-0.966485
13	1	0	-1.471995	-1.505154	-1.714217
14	6	0	-2.697261	-0.060361	-0.923182
15	1	0	-3.520706	-0.268186	-1.591577
16	17	0	-3.048157	1.220541	0.194490
17	17	0	-0.232749	-1.892070	1.061707
18	1	0	-0.498713	0.378143	0.532112

$C_8H_{12}Cl_4$ in water

opt freq wb97xd scrf=(smd,solvent=water) def2tzvp

Standard orientation:

Center Number	Atomic Number	Atomic Type	Coordinates (Angstroms) X	Y	Z
1	6	0	3.841613	0.058513	-0.394171
2	6	0	2.833987	-0.437180	0.296089
3	1	0	2.914601	-1.420932	0.747590
4	6	0	1.513806	0.221718	0.541052
5	1	0	1.381454	0.341859	1.616125
6	17	0	1.447812	1.906699	-0.111288
7	6	0	0.377463	-0.614415	-0.032581
8	1	0	0.499586	-1.633731	0.343261
9	1	0	0.481558	-0.655570	-1.119791
10	6	0	-1.026826	-0.131221	0.276934
11	6	0	-2.071036	-1.007670	-0.387652
12	1	0	-1.975582	-2.029039	-0.008434
13	1	0	-1.840779	-1.038941	-1.456293
14	6	0	-3.517728	-0.591792	-0.205555
15	1	0	-3.750312	-0.462519	0.848957
16	17	0	-3.781678	1.067620	-0.918839
17	6	0	-4.481395	-1.564870	-0.844188
18	1	0	-4.358273	-2.541821	-0.371709
19	1	0	-4.282608	-1.670673	-1.911971
20	17	0	5.325652	-0.816992	-0.613759
21	1	0	3.848821	1.025998	-0.873940
22	1	0	-1.152118	0.906869	-0.022620
23	17	0	-1.290156	-0.108833	2.080062
24	1	0	-5.513385	-1.244388	-0.705745

$C_8H_{12}Cl_4$ in acetonitrile

opt freq wb97xd scrf=(smd,solvent= acetonitrile) def2tzvp

Standard orientation:

Center Number	Atomic Number	Atomic Type	Coordinates (Angstroms) X	Y	Z
1	6	0	3.840779	0.069896	-0.398960

2	6	0	2.835041	-0.440964	0.283303
3	1	0	2.917151	-1.435356	0.711248
4	6	0	1.514869	0.211500	0.548551
5	1	0	1.380606	0.293086	1.627095
6	17	0	1.452715	1.920028	-0.041323
7	6	0	0.377904	-0.601121	-0.058421
8	1	0	0.504697	-1.637180	0.267365
9	1	0	0.478076	-0.590176	-1.146900
10	6	0	-1.027640	-0.138651	0.279244
11	6	0	-2.071474	-0.985039	-0.425370
12	1	0	-1.975060	-2.022664	-0.092932
13	1	0	-1.840536	-0.968265	-1.494349
14	6	0	-3.519374	-0.578713	-0.227051
15	1	0	-3.750762	-0.482563	0.831442
16	17	0	-3.787879	1.102022	-0.888207
17	6	0	-4.482967	-1.534309	-0.893005
18	1	0	-4.361362	-2.523729	-0.446041
19	1	0	-4.283837	-1.612195	-1.963400
20	17	0	5.323615	-0.801599	-0.645305
21	1	0	3.847648	1.048496	-0.856091
22	1	0	-1.157578	0.912915	0.032698
23	17	0	-1.285309	-0.207552	2.082312
24	1	0	-5.515270	-1.217249	-0.746978

$C_{10}H_{15}Cl_5$ in water

opt freq wb97xd scrf=(smd,solvent=water) def2tzvp

Standard orientation:

Center Number	Atomic Number	Atomic Type	Coordinates (Angstroms) X	Y	Z
1	6	0	4.703356	0.046919	0.446495
2	6	0	3.317777	0.065383	1.061213
3	1	0	3.156438	1.032063	1.546699
4	1	0	3.304176	-0.695193	1.847161
5	6	0	2.148704	-0.167593	0.123587
6	1	0	2.171749	0.542289	-0.700196
7	17	0	2.308178	-1.793795	-0.682652
8	6	0	0.828466	-0.087394	0.865597
9	1	0	0.800135	0.877495	1.378963
10	1	0	0.803974	-0.862824	1.635999

11	6	0	-0.426905	-0.242170	0.029191
12	6	0	-1.669209	-0.221835	0.899196
13	1	0	-1.724187	0.731567	1.432116
14	1	0	-1.556728	-1.007030	1.651449
15	6	0	-2.994017	-0.405440	0.180321
16	1	0	-3.112791	0.332311	-0.608193
17	17	0	-3.032094	-2.010765	-0.687708
18	6	0	-4.139772	-0.372936	1.129171
19	1	0	-4.114215	-1.083976	1.948597
20	6	0	-5.171792	0.451115	1.087539
21	17	0	4.837484	1.341695	-0.833064
22	1	0	4.870587	-0.879164	-0.098629
23	1	0	-0.387039	-1.157019	-0.557701
24	17	0	-0.515439	1.084152	-1.215439
25	17	0	-5.405726	1.666778	-0.129584
26	6	0	5.788705	0.256838	1.477403
27	1	0	5.735474	-0.547528	2.214256
28	1	0	6.776219	0.236482	1.017738
29	1	0	5.657213	1.208594	1.994700
30	1	0	-5.963723	0.437504	1.822360

$C_{10}H_{15}Cl_5$ in acetonitrile

opt freq wb97xd scrf=(smd,solvent= acetonitrile) def2tzvp

Standard orientation:

Center Number	Atomic Number	Atomic Type	Coordinates (Angstroms) X	Y	Z
1	6	0	4.706018	0.046529	0.444254
2	6	0	3.319626	0.061073	1.058901
3	1	0	3.157028	1.026568	1.546720
4	1	0	3.307249	-0.701233	1.843466
5	6	0	2.149882	-0.171902	0.120964
6	1	0	2.173652	0.537458	-0.703339
7	17	0	2.308167	-1.799070	-0.684323
8	6	0	0.828760	-0.089822	0.862749
9	1	0	0.802889	0.873796	1.379064
10	1	0	0.802116	-0.867362	1.631203
11	6	0	-0.427648	-0.238387	0.025412
12	6	0	-1.670504	-0.224392	0.896063
13	1	0	-1.727778	0.727328	1.432206
14	1	0	-1.555714	-1.011660	1.646109
15	6	0	-2.996062	-0.409518	0.177463
16	1	0	-3.115262	0.327552	-0.611687

17	17	0	-3.034062	-2.015851	-0.689732
18	6	0	-4.142386	-0.376241	1.126468
19	1	0	-4.118994	-1.089636	1.944252
20	6	0	-5.173204	0.449797	1.087343
21	17	0	4.840990	1.348805	-0.828120
22	1	0	4.873602	-0.876596	-0.106110
23	1	0	-0.387892	-1.149143	-0.567962
24	17	0	-0.516347	1.096918	-1.209986
25	17	0	-5.407367	1.670117	-0.125487
26	6	0	5.792062	0.249777	1.476690
27	1	0	5.739866	-0.559464	2.208725
28	1	0	6.779625	0.232210	1.016306
29	1	0	5.661764	1.198504	2.000382
30	1	0	-5.964886	0.434555	1.822866

$C_{12}H_{18}Cl_6$ in water

opt freq wb97xd scrf=(smd,solvent=water) def2tzvp

Standard orientation:

Center Number	Atomic Number	Atomic Type	Coordinates (Angstroms) X	Y	Z
1	6	0	4.243764	0.370477	0.341944
2	6	0	5.339033	0.244848	1.340976
3	1	0	5.210072	0.779781	2.276383
4	6	0	2.884634	0.117735	0.968817
5	1	0	2.718120	0.843161	1.769316
6	1	0	2.922920	-0.872126	1.432159
7	6	0	1.694334	0.188399	0.031639
8	1	0	1.672932	1.143513	-0.488414
9	17	0	1.877818	-1.041754	-1.298590
10	6	0	0.395343	-0.044732	0.778683
11	1	0	0.353201	0.683203	1.593600
12	1	0	0.418347	-1.038081	1.234862
13	6	0	-0.878007	0.048601	-0.039440
14	6	0	-2.097847	-0.273819	0.802339
15	1	0	-2.163469	0.442695	1.625809
16	1	0	-1.936527	-1.260258	1.245672
17	6	0	-3.434722	-0.261411	0.086617
18	1	0	-3.586002	0.685070	-0.427218
19	17	0	-3.434508	-1.499085	-1.251438
20	6	0	-4.577088	-0.531278	1.046684
21	1	0	-4.507088	0.210637	1.847267
22	1	0	-4.438774	-1.514242	1.506038

23	6	0	-5.978327	-0.499897	0.468376
24	6	0	-7.034931	-0.787934	1.509987
25	1	0	-6.986106	-0.065390	2.326201
26	1	0	-8.032797	-0.762889	1.073490
27	1	0	-6.863677	-1.785464	1.920614
28	6	0	6.444655	-0.467097	1.210720
29	1	0	7.196577	-0.530653	1.983969
30	17	0	6.853425	-1.393955	-0.198860
31	17	0	4.313174	2.054663	-0.362966
32	1	0	4.408337	-0.284176	-0.508958
33	1	0	-0.826215	-0.606801	-0.905877
34	17	0	-1.035434	1.717788	-0.752816
35	1	0	-6.066992	-1.192243	-0.365651
36	17	0	-6.309647	1.132985	-0.277878

$C_{12}H_{18}Cl_6$ in acetonitrile

opt freq wb97xd scrf=(smd,solvent= acetonitrile) def2tzvp

Standard orientation:

Center Number	Atomic Number	Atomic Type	Coordinates (Angstroms) X	Y	Z
1	6	0	4.245726	0.376187	0.338365
2	6	0	5.342226	0.257425	1.337694
3	1	0	5.213944	0.798549	2.269945
4	6	0	2.886262	0.125402	0.967096
5	1	0	2.717048	0.859086	1.759773
6	1	0	2.927754	-0.859279	1.441577
7	6	0	1.695516	0.181500	0.028302
8	1	0	1.674723	1.128629	-0.506382
9	17	0	1.879341	-1.069372	-1.282957
10	6	0	0.395025	-0.039609	0.777894
11	1	0	0.355393	0.696079	1.586184
12	1	0	0.414595	-1.028824	1.243549
13	6	0	-0.878877	0.051210	-0.041156
14	6	0	-2.099835	-0.268843	0.801261
15	1	0	-2.167724	0.452621	1.620463
16	1	0	-1.937087	-1.252373	1.250971
17	6	0	-3.436922	-0.264120	0.084218
18	1	0	-3.587743	0.677132	-0.439374
19	17	0	-3.435979	-1.515895	-1.240899
20	6	0	-4.580613	-0.523681	1.046740
21	1	0	-4.508467	0.223681	1.842285
22	1	0	-4.444758	-1.503947	1.513050

23	6	0	-5.982742	-0.492409	0.468784
24	6	0	-7.040248	-0.776898	1.511333
25	1	0	-6.991859	-0.052355	2.326159
26	1	0	-8.038162	-0.751867	1.074194
27	1	0	-6.871007	-1.773964	1.924717
28	6	0	6.448968	-0.453991	1.211761
29	1	0	7.201161	-0.510739	1.985720
30	17	0	6.860511	-1.389938	-0.191360
31	17	0	4.313604	2.056113	-0.377681
32	1	0	4.410870	-0.283802	-0.508373
33	1	0	-0.826777	-0.607217	-0.905366
34	17	0	-1.035845	1.718298	-0.760116
35	1	0	-6.072457	-1.187499	-0.363090
36	17	0	-6.313183	1.138620	-0.282855

$C_{14}H_{21}Cl_7$ in water

opt freq wb97xd scrf=(smd,solvent=water) def2tzvp

Standard orientation:

Center Number	Atomic Number	Atomic Type	Coordinates (Angstroms) X	Y	Z
1	6	0	-2.986930	-0.211803	0.022909
2	1	0	-3.051917	-1.180349	-0.467563
3	6	0	-4.138795	-0.026853	0.992179
4	1	0	-4.098114	0.979874	1.417041
5	1	0	-3.993522	-0.731756	1.814799
6	17	0	-3.122782	0.990093	-1.338872
7	6	0	-1.651691	-0.053356	0.723758
8	1	0	-1.638541	-0.756158	1.561470
9	1	0	-1.586075	0.952607	1.147044
10	6	0	-0.413471	-0.268704	-0.124913
11	6	0	0.850270	-0.015109	0.674419
12	1	0	0.891581	-0.718840	1.510454
13	1	0	0.769764	0.987935	1.102095
14	6	0	2.160775	-0.131586	-0.079387
15	1	0	2.231618	-1.093868	-0.580909
16	17	0	2.205907	1.082625	-1.437771
17	6	0	3.346667	0.076771	0.842831
18	1	0	3.252999	-0.645671	1.658437
19	1	0	3.285972	1.074248	1.286498
20	6	0	4.721183	-0.056829	0.216102
21	6	0	5.819032	0.202729	1.229673
22	1	0	5.746768	-0.534509	2.034336

23	1	0	5.628657	1.183593	1.674605
24	6	0	7.243904	0.163815	0.712337
25	1	0	7.442770	-0.776250	0.202820
26	17	0	7.470073	1.424555	-0.588736
27	6	0	8.260313	0.396762	1.806193
28	1	0	8.156202	-0.388849	2.557841
29	1	0	9.275435	0.362059	1.412092
30	1	0	8.100145	1.361340	2.290745
31	6	0	-5.530337	-0.215847	0.415163
32	6	0	-6.583991	0.009886	1.440958
33	1	0	-6.472692	-0.522911	2.379813
34	6	0	-7.629490	0.810583	1.331683
35	1	0	-8.352044	0.943884	2.123850
36	17	0	-7.999482	1.754312	-0.077463
37	17	0	-5.724383	-1.916346	-0.224144
38	1	0	-5.682533	0.417065	-0.454358
39	17	0	-0.401191	-1.965817	-0.787898
40	1	0	-0.440402	0.362299	-1.010444
41	17	0	4.919431	-1.720671	-0.500941
42	1	0	4.820537	0.612746	-0.635074

$C_{14}H_{21}Cl_7$ in acetonitrile

opt freq wb97xd scrf=(smd,solvent= acetonitrile) def2tzvp

Standard orientation:

Center Number	Atomic Number	Atomic Type	Coordinates (Angstroms) X	Y	Z
1	6	0	-2.989757	-0.207846	0.016738
2	1	0	-3.054771	-1.172275	-0.481932
3	6	0	-4.140946	-0.032561	0.989851
4	1	0	-4.103637	0.972650	1.419134
5	1	0	-3.990779	-0.740423	1.809310
6	17	0	-3.129026	1.005767	-1.334314
7	6	0	-1.652880	-0.055072	0.717329
8	1	0	-1.639072	-0.764544	1.549629
9	1	0	-1.586942	0.947662	1.148697
10	6	0	-0.414130	-0.264042	-0.133538
11	6	0	0.850259	-0.016106	0.668003
12	1	0	0.892323	-0.728024	1.497272
13	1	0	0.768479	0.982758	1.105638
14	6	0	2.162007	-0.123421	-0.086520
15	1	0	2.232184	-1.079250	-0.600508
16	17	0	2.210266	1.108739	-1.428502

17	6	0	3.347884	0.071030	0.840063
18	1	0	3.249750	-0.658259	1.649291
19	1	0	3.290438	1.064908	1.292644
20	6	0	4.723857	-0.062980	0.214808
21	6	0	5.821407	0.190972	1.231227
22	1	0	5.747800	-0.550460	2.032203
23	1	0	5.630829	1.169677	1.681277
24	6	0	7.247885	0.154841	0.716136
25	1	0	7.447304	-0.782083	0.200693
26	17	0	7.477990	1.424316	-0.576206
27	6	0	8.263334	0.379078	1.813544
28	1	0	8.157734	-0.411379	2.560358
29	1	0	9.279219	0.345308	1.420485
30	1	0	8.104814	1.341036	2.304401
31	6	0	-5.533848	-0.225303	0.415419
32	6	0	-6.587080	-0.001059	1.442737
33	1	0	-6.476678	-0.537732	2.379810
34	6	0	-7.631984	0.801364	1.337663
35	1	0	-8.353615	0.931373	2.131657
36	17	0	-8.004056	1.752436	-0.066364
37	17	0	-5.725560	-1.926781	-0.223132
38	1	0	-5.688852	0.407071	-0.454067
39	17	0	-0.401541	-1.956175	-0.809649
40	1	0	-0.441195	0.373985	-1.014100
41	17	0	4.919745	-1.724503	-0.508845
42	1	0	4.825734	0.610037	-0.633451

$C_4H_7Cl_3$ in water

opt freq wb97xd scrf=(smd,solvent=water) def2tzvp

Standard orientation:

Center Number	Atomic Number	Atomic Type	Coordinates (Angstroms) X	Y	Z
1	6	0	0.706983	0.476988	0.280676
2	1	0	0.767130	0.385389	1.363760
3	6	0	2.010210	0.029697	-0.355722
4	1	0	1.951615	0.057265	-1.441453
5	1	0	2.840324	0.638148	-0.010147
6	17	0	0.590306	2.261606	-0.033812
7	6	0	-0.516912	-0.237266	-0.262827
8	1	0	-0.350534	-1.308197	-0.129166
9	1	0	-0.609308	-0.044787	-1.333512
10	6	0	-1.784505	0.158717	0.462609

11	1	0	-2.044583	1.201046	0.300641
12	17	0	-3.185586	-0.801693	-0.135213
13	1	0	-1.710660	-0.035991	1.530226
14	17	0	2.398889	-1.663542	0.108509

$C_4H_7Cl_3$ in acetonitrile

opt freq wb97xd scrf=(smd,solvent= acetonitrile) def2tzvp

Standard orientation:

Center Number	Atomic Number	Atomic Type	Coordinates (Angstroms) X	Y	Z
1	6	0	0.707120	0.477257	0.279679
2	1	0	0.766960	0.384985	1.362986
3	6	0	2.010746	0.027657	-0.355783
4	1	0	1.953698	0.055171	-1.441873
5	1	0	2.841314	0.635754	-0.009797
6	17	0	0.594203	2.263013	-0.033368
7	6	0	-0.518445	-0.235051	-0.264373
8	1	0	-0.352378	-1.306555	-0.133437
9	1	0	-0.611999	-0.040446	-1.334795
10	6	0	-1.786371	0.159125	0.462761
11	1	0	-2.047365	1.201792	0.302920
12	17	0	-3.188358	-0.800873	-0.134950
13	1	0	-1.711934	-0.037186	1.530366
14	17	0	2.399061	-1.666107	0.108902

$C_6H_{10}Cl_4$ in water

opt freq wb97xd scrf=(smd,solvent=water) def2tzvp

Standard orientation:

Center Number	Atomic Number	Atomic Type	Coordinates (Angstroms) X	Y	Z
1	6	0	1.655231	0.515310	0.187274
2	1	0	1.693822	0.143736	1.208948
3	6	0	3.040126	0.473659	-0.431702
4	1	0	3.020124	0.777826	-1.475736
5	1	0	3.734163	1.094201	0.126568
6	17	0	1.228450	2.272008	0.342724
7	6	0	0.604298	-0.207335	-0.631068

8	1	0	0.947965	-1.231833	-0.792532
9	1	0	0.532157	0.268118	-1.612878
10	6	0	-0.790223	-0.242854	-0.036050
11	6	0	-1.777399	-0.905116	-0.978713
12	1	0	-1.468572	-1.937928	-1.163287
13	1	0	-1.717784	-0.376605	-1.933273
14	6	0	-3.214228	-0.943909	-0.514488
15	1	0	-3.839599	-1.439859	-1.251238
16	1	0	-3.326814	-1.440334	0.445728
17	17	0	-3.910712	0.710359	-0.304002
18	17	0	-0.760761	-1.123933	1.557588
19	1	0	-1.124895	0.760385	0.220060
20	17	0	3.704353	-1.197036	-0.381244

$C_6H_{10}Cl_4$ in acetonitrile

opt freq wb97xd scrf=(smd,solvent= acetonitrile) def2tzvp

Standard orientation:

Center Number	Atomic Number	Atomic Type	Coordinates (Angstroms) X	Y	Z
1	6	0	1.655439	0.515816	0.189699
2	1	0	1.696184	0.140332	1.210023
3	6	0	3.040297	0.478461	-0.431121
4	1	0	3.019599	0.788668	-1.473635
5	1	0	3.733960	1.097514	0.129819
6	17	0	1.227415	2.272153	0.353018
7	6	0	0.603360	-0.205207	-0.629679
8	1	0	0.949167	-1.228152	-0.797213
9	1	0	0.527882	0.275013	-1.609116
10	6	0	-0.790497	-0.247355	-0.031676
11	6	0	-1.780157	-0.902456	-0.977863
12	1	0	-1.474933	-1.935847	-1.166728
13	1	0	-1.718781	-0.370515	-1.930675
14	6	0	-3.218199	-0.938686	-0.515211
15	1	0	-3.843009	-1.434450	-1.253082
16	1	0	-3.333458	-1.435114	0.444922
17	17	0	-3.914367	0.716359	-0.306417
18	17	0	-0.756956	-1.141600	1.554896
19	1	0	-1.125306	0.753577	0.233487
20	17	0	3.709039	-1.191292	-0.390479

$C_8H_{13}Cl_5$ in water

opt freq wb97xd scrf=(smd,solvent=water) def2tzvp

Standard orientation:

Center Number	Atomic Number	Atomic Type	Coordinates (Angstroms) X	Y	Z
1	6	0	4.605071	-0.231965	-0.879975
2	6	0	3.170681	-0.452134	-1.299083
3	1	0	2.989961	-1.521824	-1.430430
4	1	0	3.042528	0.018405	-2.278065
5	6	0	2.111677	0.069675	-0.345662
6	1	0	2.269371	-0.331616	0.653250
7	17	0	2.296113	1.870114	-0.136981
8	6	0	0.715195	-0.254545	-0.839645
9	1	0	0.665004	-1.336418	-0.990453
10	1	0	0.563240	0.213663	-1.816176
11	6	0	-0.435985	0.176490	0.048534
12	6	0	-1.771718	-0.160180	-0.586307
13	1	0	-1.837992	-1.241888	-0.724784
14	1	0	-1.788099	0.294184	-1.580540
15	6	0	-3.001546	0.287727	0.177561
16	1	0	-2.984146	-0.061138	1.207573
17	17	0	-3.039844	2.097967	0.297895
18	6	0	-4.304356	-0.117845	-0.486632
19	1	0	-4.317318	0.153058	-1.540040
20	17	0	5.002082	-1.078994	0.666458
21	1	0	4.830515	0.818047	-0.713307
22	1	0	-0.371971	1.239838	0.267991
23	17	0	-0.302627	-0.625549	1.677952
24	1	0	-5.156577	0.320845	0.022731
25	1	0	5.287632	-0.633122	-1.623587
26	17	0	-4.527857	-1.900914	-0.398672

$C_8H_{13}Cl_5$ in acetonitrile

opt freq wb97xd scrf=(smd,solvent= acetonitrile) def2tzvp

Standard orientation:

Center Number	Atomic Number	Atomic Type	Coordinates (Angstroms) X	Y	Z
1	6	0	4.608576	-0.231027	-0.877155
2	6	0	3.173526	-0.448776	-1.297381

3	1	0	2.991750	-1.518601	-1.428159
4	1	0	3.047755	0.020892	-2.277402
5	6	0	2.113246	0.075976	-0.345936
6	1	0	2.271009	-0.321681	0.654489
7	17	0	2.296297	1.877630	-0.143453
8	6	0	0.716230	-0.251367	-0.838691
9	1	0	0.668613	-1.333245	-0.991534
10	1	0	0.562023	0.218133	-1.814446
11	6	0	-0.435686	0.174613	0.052251
12	6	0	-1.772195	-0.156032	-0.585816
13	1	0	-1.839498	-1.236791	-0.732027
14	1	0	-1.787318	0.305344	-1.577042
15	6	0	-3.003084	0.287466	0.180074
16	1	0	-2.985504	-0.066600	1.208452
17	17	0	-3.043614	2.097537	0.309751
18	6	0	-4.306412	-0.116033	-0.485752
19	1	0	-4.321207	0.161865	-1.537600
20	17	0	5.006549	-1.082455	0.667010
21	1	0	4.835652	0.818507	-0.708348
22	1	0	-0.370666	1.236422	0.279034
23	17	0	-0.303298	-0.638745	1.676420
24	1	0	-5.158557	0.319453	0.027101
25	1	0	5.290566	-0.631078	-1.622406
26	17	0	-4.530630	-1.899940	-0.409120

$C_{10}H_{16}Cl_6$ in water

opt freq wb97xd scrf=(smd,solvent=water) def2tzvp

Standard orientation:

Center Number	Atomic Number	Atomic Type	Coordinates (Angstroms) X	Y	Z
1	6	0	-4.267382	0.357497	-0.002946
2	6	0	-2.972958	-0.138369	-0.615235
3	1	0	-2.861383	0.302904	-1.609469
4	1	0	-3.055544	-1.219446	-0.749391
5	6	0	-1.709083	0.169198	0.165339
6	1	0	-1.644590	1.231290	0.390339
7	17	0	-1.781157	-0.631000	1.799627
8	6	0	-0.476588	-0.290447	-0.589494
9	1	0	-0.499087	0.193076	-1.569862
10	1	0	-0.542500	-1.368592	-0.760227

11	6	0	0.862299	-0.018351	0.068271
12	6	0	2.004235	-0.555749	-0.772770
13	1	0	1.990218	-0.065350	-1.749940
14	1	0	1.811500	-1.618587	-0.942694
15	6	0	3.400525	-0.388009	-0.205396
16	1	0	3.594788	0.655837	0.032155
17	17	0	3.530337	-1.241632	1.398769
18	6	0	4.453112	-0.918659	-1.160999
19	1	0	4.301355	-0.417061	-2.119975
20	1	0	4.288576	-1.986927	-1.327930
21	6	0	5.889946	-0.749314	-0.726945
22	17	0	-4.259919	2.169152	0.097126
23	1	0	-4.381683	0.020075	1.024826
24	1	0	0.890874	-0.441060	1.069786
25	17	0	1.074020	1.772247	0.331813
26	1	0	6.090117	-1.216241	0.233796
27	17	0	6.348495	0.989143	-0.541880
28	6	0	-5.498790	-0.010812	-0.809752
29	1	0	-6.388115	0.455414	-0.397486
30	1	0	-5.384591	0.257622	-1.857495
31	1	0	6.565608	-1.157311	-1.473149
32	17	0	-5.787506	-1.785413	-0.749793

$C_{10}H_{16}Cl_6$ in acetonitrile

opt freq wb97xd scrf=(smd,solvent= acetonitrile) def2tzvp

Standard orientation:

Center Number	Atomic Number	Atomic Type	Coordinates (Angstroms) X	Y	Z
1	6	0	-4.269547	0.358641	0.000132
2	6	0	-2.974470	-0.132794	-0.615869
3	1	0	-2.861675	0.317699	-1.606040
4	1	0	-3.058411	-1.212571	-0.760318
5	6	0	-1.710048	0.165709	0.168779
6	1	0	-1.645733	1.225344	0.405502
7	17	0	-1.782675	-0.652554	1.794403
8	6	0	-0.476306	-0.285319	-0.590752
9	1	0	-0.500772	0.204803	-1.568003
10	1	0	-0.539665	-1.362612	-0.768948

11	6	0	0.863170	-0.013951	0.067859
12	6	0	2.006362	-0.550953	-0.773017
13	1	0	1.995185	-0.057010	-1.748662
14	1	0	1.811805	-1.612965	-0.947133
15	6	0	3.402795	-0.388569	-0.202439
16	1	0	3.597827	0.653638	0.041957
17	17	0	3.529559	-1.252616	1.396777
18	6	0	4.456827	-0.914469	-1.160166
19	1	0	4.304656	-0.410376	-2.118025
20	1	0	4.294055	-1.982748	-1.330525
21	6	0	5.894304	-0.744648	-0.726417
22	17	0	-4.262637	2.169961	0.114326
23	1	0	-4.383453	0.013253	1.025460
24	1	0	0.891175	-0.438033	1.068871
25	17	0	1.074784	1.776547	0.333837
26	1	0	6.095152	-1.211363	0.234534
27	17	0	6.354998	0.993813	-0.543132
28	6	0	-5.502300	-0.003731	-0.808480
29	1	0	-6.391196	0.458983	-0.390603
30	1	0	-5.390632	0.274743	-1.854156
31	1	0	6.569483	-1.153864	-1.472904
32	17	0	-5.792412	-1.779059	-0.764964

$C_{12}H_{19}Cl_7$ in water

opt freq wb97xd scrf=(smd,solvent=water) def2tzvp

Standard orientation:

Center Number	Atomic Number	Atomic Type	Coordinates (Angstroms) X	Y	Z
1	6	0	-4.694204	-0.058649	-0.468304
2	6	0	-5.731531	-0.599391	-1.434663
3	1	0	-5.534132	-1.667007	-1.558823
4	1	0	-5.596117	-0.131016	-2.413728
5	6	0	-3.285375	-0.363698	-0.939101
6	1	0	-3.125087	0.105354	-1.913878
7	1	0	-3.216941	-1.444881	-1.087407
8	6	0	-2.155980	0.086237	-0.032486
9	1	0	-2.245388	1.146812	0.191048

10	17	0	-2.300595	-0.725145	1.592173
11	6	0	-0.803626	-0.218688	-0.647208
12	1	0	-0.782300	0.248613	-1.635393
13	1	0	-0.713284	-1.297798	-0.799453
14	6	0	0.412238	0.229446	0.141022
15	6	0	1.697536	-0.194453	-0.543340
16	1	0	1.737956	0.252727	-1.540314
17	1	0	1.651954	-1.278677	-0.677993
18	6	0	2.989732	0.167765	0.162570
19	1	0	3.032512	1.237839	0.352847
20	17	0	3.032898	-0.583097	1.821309
21	6	0	4.197923	-0.283690	-0.636661
22	1	0	4.093768	0.116018	-1.648958
23	1	0	4.185101	-1.373204	-0.717078
24	6	0	5.546047	0.132008	-0.083363
25	6	0	6.719883	-0.404227	-0.881497
26	1	0	6.604513	-0.205545	-1.944604
27	1	0	7.656753	0.009214	-0.521432
28	6	0	-7.174744	-0.397749	-1.036907
29	1	0	-7.416994	0.649579	-0.877205
30	1	0	-7.841259	-0.810964	-1.788414
31	17	0	-7.581732	-1.244606	0.507116
32	17	0	-4.908026	1.740009	-0.270825
33	1	0	-4.860982	-0.457678	0.530019
34	1	0	0.373926	-0.156098	1.157071
35	17	0	0.383759	2.039471	0.349114
36	1	0	5.654221	-0.140910	0.964000
37	17	0	5.702781	1.939657	-0.113158
38	17	0	6.849409	-2.188161	-0.689885

$C_{12}H_{19}Cl_7$ in acetonitrile

opt freq wb97xd scrf=(smd,solvent= acetonitrile) def2tzvp

Standard orientation:

Center Number	Atomic Number	Atomic Type	Coordinates (Angstroms) X	Y	Z
1	6	0	-4.697345	-0.051070	-0.468731
2	6	0	-5.736856	-0.592175	-1.433624
3	1	0	-5.539902	-1.660042	-1.558432
4	1	0	-5.603244	-0.123814	-2.413254
5	6	0	-3.288461	-0.360008	-0.939256
6	1	0	-3.125541	0.112372	-1.912173

7	1	0	-3.223952	-1.441002	-1.092011
8	6	0	-2.157676	0.081647	-0.029073
9	1	0	-2.246845	1.140326	0.203658
10	17	0	-2.301751	-0.743968	1.588719
11	6	0	-0.804707	-0.217714	-0.647051
12	1	0	-0.785402	0.253934	-1.633418
13	1	0	-0.712636	-1.296168	-0.804260
14	6	0	0.411990	0.229733	0.141808
15	6	0	1.698246	-0.193484	-0.542796
16	1	0	1.740946	0.258860	-1.537550
17	1	0	1.650999	-1.277059	-0.683343
18	6	0	2.990700	0.162357	0.167498
19	1	0	3.031756	1.230362	0.369778
20	17	0	3.034496	-0.607088	1.817877
21	6	0	4.200371	-0.277435	-0.637368
22	1	0	4.094945	0.133946	-1.645060
23	1	0	4.189994	-1.366087	-0.730754
24	6	0	5.548903	0.133959	-0.080170
25	6	0	6.724062	-0.391419	-0.884744
26	1	0	6.608618	-0.181967	-1.946022
27	1	0	7.660180	0.020932	-0.520676
28	6	0	-7.180414	-0.391380	-1.034432
29	1	0	-7.422847	0.655609	-0.871382
30	1	0	-7.846981	-0.801691	-1.787999
31	17	0	-7.589409	-1.243908	0.506340
32	17	0	-4.907421	1.748707	-0.274604
33	1	0	-4.864368	-0.447803	0.530546
34	1	0	0.373554	-0.157276	1.157373
35	17	0	0.383654	2.039796	0.352174
36	1	0	5.657994	-0.151427	0.963858
37	17	0	5.703606	1.942715	-0.087672
38	17	0	6.859757	-2.177434	-0.712317

$C_{14}H_{22}Cl_8$ in water

opt freq wb97xd scrf=(smd,solvent=water) def2tzvp

Standard orientation:

Center Number	Atomic Number	Atomic Type	Coordinates (Angstroms) X	Y	Z
1	6	0	3.442527	-0.093719	0.035943
2	1	0	3.527578	-0.494041	1.043514
3	6	0	4.537105	-0.649526	-0.854880
4	1	0	4.468636	-0.180171	-1.840063

5	1	0	4.336313	-1.715676	-0.991574
6	17	0	3.666194	1.702031	0.248261
7	6	0	2.069654	-0.379750	-0.541123
8	1	0	1.996665	-1.460964	-0.687691
9	1	0	1.991287	0.084716	-1.527969
10	6	0	0.879362	0.091933	0.271950
11	6	0	-0.425776	-0.223395	-0.433202
12	1	0	-0.516777	-1.306371	-0.553681
13	1	0	-0.367004	0.207677	-1.436320
14	6	0	-1.691179	0.265198	0.244979
15	1	0	-1.729437	-0.075631	1.276887
16	17	0	-1.662634	2.082201	0.377817
17	6	0	-2.928757	-0.180107	-0.509925
18	1	0	-2.878684	-1.268539	-0.603057
19	1	0	-2.900104	0.231286	-1.522622
20	6	0	-4.264357	0.207006	0.094948
21	6	0	-5.416668	-0.285182	-0.760305
22	1	0	-5.391107	-1.376875	-0.795308
23	1	0	-5.251734	0.074186	-1.779529
24	6	0	-6.800070	0.138921	-0.309509
25	1	0	-6.969815	-0.089357	0.740428
26	17	0	-6.968439	1.941777	-0.428380
27	6	0	-7.918208	-0.442508	-1.154695
28	1	0	-8.878994	-0.026432	-0.868115
29	1	0	-7.741682	-0.281567	-2.215786
30	6	0	5.961866	-0.470351	-0.367980
31	6	0	6.962345	-1.020444	-1.367288
32	1	0	6.760065	-0.537143	-2.326322
33	1	0	6.789580	-2.091603	-1.504874
34	6	0	8.420381	-0.843681	-1.014396
35	1	0	8.671657	-1.291832	-0.056806
36	1	0	9.054633	-1.267814	-1.787457
37	17	0	8.888537	0.897427	-0.889426
38	17	0	6.178036	-1.290977	1.244390
39	1	0	6.167103	0.578142	-0.162300
40	17	0	0.899280	-0.686128	1.919223
41	1	0	0.955961	1.156802	0.479437
42	17	0	-4.413563	-0.476510	1.776808
43	1	0	-4.322154	1.283700	0.238729
44	17	0	-8.042784	-2.219167	-0.903200

$C_{14}H_{22}Cl_8$ in acetonitrile

opt freq wb97xd scrf=(smd,solvent= acetonitrile) def2tzvp

Standard orientation:

Center Number	Atomic Number	Atomic Type	Coordinates (Angstroms) X	Y	Z
1	6	0	3.445347	-0.088358	0.037452
2	1	0	3.531913	-0.485817	1.046099
3	6	0	4.540144	-0.646190	-0.853144
4	1	0	4.472830	-0.176347	-1.838386
5	1	0	4.337619	-1.712115	-0.990460
6	17	0	3.667707	1.708374	0.244376
7	6	0	2.071298	-0.377796	-0.537114
8	1	0	2.000817	-1.459274	-0.684453
9	1	0	1.990003	0.087096	-1.523750
10	6	0	0.880241	0.089774	0.278584
11	6	0	-0.425484	-0.221530	-0.428925
12	1	0	-0.517453	-1.304217	-0.553047
13	1	0	-0.365134	0.212687	-1.430814
14	6	0	-1.692130	0.266381	0.249064
15	1	0	-1.731574	-0.077177	1.280100
16	17	0	-1.663341	2.083261	0.386507
17	6	0	-2.929814	-0.176722	-0.508537
18	1	0	-2.876903	-1.264338	-0.611112
19	1	0	-2.903071	0.243301	-1.517931
20	6	0	-4.266665	0.201353	0.101141
21	6	0	-5.419157	-0.275232	-0.763947
22	1	0	-5.394665	-1.366295	-0.818169
23	1	0	-5.252470	0.101833	-1.776671
24	6	0	-6.803802	0.142262	-0.308802
25	1	0	-6.974510	-0.099704	0.738061
26	17	0	-6.973705	1.947083	-0.403730
27	6	0	-7.921675	-0.429397	-1.162105
28	1	0	-8.882630	-0.015468	-0.871948
29	1	0	-7.744380	-0.257297	-2.221582
30	6	0	5.965915	-0.470103	-0.365824
31	6	0	6.966349	-1.017273	-1.367823
32	1	0	6.762426	-0.532791	-2.326183
33	1	0	6.794949	-2.088701	-1.507144
34	6	0	8.425531	-0.839536	-1.017619
35	1	0	8.679133	-1.286904	-0.060032
36	1	0	9.058139	-1.265180	-1.791696
37	17	0	8.895537	0.901658	-0.896124
38	17	0	6.182123	-1.296732	1.243826
39	1	0	6.171934	0.577627	-0.156588
40	17	0	0.899967	-0.696684	1.922053
41	1	0	0.956850	1.153678	0.491269
42	17	0	-4.418046	-0.508806	1.771897
43	1	0	-4.323566	1.275804	0.261642

44	17	0	-8.049469	-2.209108	-0.930664

OOH in water

opt freq wb97xd scrf=(smd,solvent=water) def2tzvp

Standard orientation:

Center Number	Atomic Number	Atomic Type	Coordinates (Angstroms) X	Y	Z
1	8	0	0.054938	-0.597523	0.000000
2	1	0	-0.879013	-0.876674	0.000000
3	8	0	0.054938	0.707108	0.000000

OOH in acetonitrile

opt freq wb97xd scrf=(smd,solvent= acetonitrile) def2tzvp

Standard orientation:

Center Number	Atomic Number	Atomic Type	Coordinates (Angstroms) X	Y	Z
1	8	0	0.055217	-0.599119	0.000000
2	1	0	-0.883479	-0.872969	0.000000
3	8	0	0.055217	0.708241	0.000000

Histidine in water

opt freq wb97xd scrf=(smd,solvent=water) def2tzvp

Standard orientation:

Center Number	Atomic Number	Atomic Type	Coordinates (Angstroms) X	Y	Z
1	8	0	1.003527	1.508135	-1.172167
2	8	0	2.080905	1.349982	0.776634
3	7	0	-1.495736	0.493588	0.859848
4	7	0	2.754062	-1.270936	-0.071980
5	7	0	-3.072740	-0.149329	-0.534871
6	6	0	0.323343	-1.219195	0.540375

7	6	0	1.457114	-0.677412	-0.358568
8	6	0	-1.021529	-0.646703	0.261430
9	6	0	1.564892	0.820270	-0.181433
10	6	0	-2.013377	-1.025866	-0.594983
11	6	0	-2.717910	0.750777	0.351784
12	1	0	0.597934	-1.044757	1.583921
13	1	0	0.288783	-2.297881	0.383871
14	1	0	1.200883	-0.877367	-1.397591
15	1	0	-1.007739	1.045299	1.548034
16	1	0	-2.019737	-1.884114	-1.247964
17	1	0	2.673317	-2.275135	-0.182864
18	1	0	2.979005	-1.109186	0.904393
19	1	0	-3.305137	1.598721	0.665492
20	1	0	1.062941	2.455002	-0.975644

Histidine in acetonitrile

opt freq wb97xd scrf=(smd,solvent= acetonitrile) def2tzvp

Standard orientation:

Center Number	Atomic Number	Atomic Type	Coordinates (Angstroms) X	Y	Z
1	8	0	0.967510	1.507755	-1.152515
2	8	0	2.132430	1.353798	0.743582
3	7	0	-1.509410	0.476632	0.877426
4	7	0	2.751062	-1.272474	-0.075339
5	7	0	-3.065684	-0.137816	-0.545680
6	6	0	0.321120	-1.218696	0.545219
7	6	0	1.455997	-0.677654	-0.358747
8	6	0	-1.027141	-0.652055	0.265797
9	6	0	1.574560	0.823096	-0.183128
10	6	0	-2.008967	-1.013471	-0.613123
11	6	0	-2.727353	0.744829	0.363189
12	1	0	0.595755	-1.040479	1.588380
13	1	0	0.291205	-2.298656	0.394545
14	1	0	1.195918	-0.879858	-1.397692
15	1	0	-1.045448	1.001085	1.602738
16	1	0	-2.008095	-1.856151	-1.287550
17	1	0	2.681022	-2.279457	-0.164069
18	1	0	3.012153	-1.078433	0.885484
19	1	0	-3.320061	1.584566	0.689809
20	1	0	1.036954	2.454265	-0.950262

Glycine in water

opt freq wb97xd scrf=(smd,solvent=water) def2tzvp

Standard orientation:

Center Number	Atomic Number	Atomic Type	Coordinates (Angstroms) X	Y	Z
1	8	0	1.637890	-0.654024	-0.001723
2	8	0	0.577066	1.312654	0.000818
3	7	0	-1.960685	0.012122	-0.001660
4	6	0	-0.720508	-0.728654	0.002320
5	6	0	0.537621	0.104173	0.000613
6	1	0	-0.670136	-1.387528	-0.866563
7	1	0	-0.671127	-1.378963	0.877809
8	1	0	-1.987026	0.617243	-0.813988
9	1	0	-1.988018	0.624078	0.805493
10	1	0	2.418776	-0.081842	-0.001492

Glycine in acetonitrile

opt freq wb97xd scrf=(smd,solvent= acetonitrile) def2tzvp

Standard orientation:

Center Number	Atomic Number	Atomic Type	Coordinates (Angstroms) X	Y	Z
1	8	0	1.635479	-0.659437	-0.000303
2	8	0	0.585924	1.311106	0.000151
3	7	0	-1.960671	0.015165	-0.000246
4	6	0	-0.722921	-0.726877	0.000466
5	6	0	0.540093	0.108271	0.000055
6	1	0	-0.677314	-1.382591	-0.872707
7	1	0	-0.677467	-1.381111	0.874781
8	1	0	-2.004822	0.618608	-0.812259
9	1	0	-2.004594	0.620553	0.810329
10	1	0	2.414639	-0.083333	-0.000328

Alanine in water

opt freq wb97xd scrf=(smd,solvent=water) def2tzvp

Standard orientation:

Center Number	Atomic Number	Atomic Type	Coordinates (Angstroms) X	Y	Z
1	8	0	-1.628773	0.768600	-0.433995
2	8	0	-1.125052	-1.123108	0.638333
3	7	0	1.533097	-1.015111	-0.344424
4	6	0	0.661826	0.141802	-0.404454
5	6	0	1.192111	1.257088	0.491320
6	6	0	-0.772365	-0.162094	-0.006872
7	1	0	0.628092	0.504048	-1.433208
8	1	0	2.200992	1.523365	0.176481
9	1	0	0.563496	2.145270	0.435010
10	1	0	1.230223	0.921986	1.530594
11	1	0	1.141865	-1.765670	-0.901597
12	1	0	1.562919	-1.356599	0.610391
13	1	0	-2.518098	0.548657	-0.121371

Alanine in acetonitrile

opt freq wb97xd scrf=(smd,solvent= acetonitrile) def2tzvp

Standard orientation:

Center Number	Atomic Number	Atomic Type	Coordinates (Angstroms) X	Y	Z
1	8	0	-1.628251	0.771769	-0.435206
2	8	0	-1.130385	-1.120146	0.636119
3	7	0	1.532243	-1.015852	-0.337288
4	6	0	0.662036	0.140624	-0.404044
5	6	0	1.196203	1.257665	0.490551
6	6	0	-0.775615	-0.164763	-0.005441
7	1	0	0.635201	0.498647	-1.435588
8	1	0	2.211736	1.509420	0.183821
9	1	0	0.578624	2.153289	0.423658
10	1	0	1.221624	0.929455	1.532818
11	1	0	1.175047	-1.758127	-0.927159
12	1	0	1.541384	-1.381770	0.608605
13	1	0	-2.515984	0.545909	-0.118833

Tyrosine in water

opt freq wb97xd scrf=(smd,solvent=water) def2tzvp

Standard orientation:

Center Number	Atomic Number	Atomic Type	Coordinates (Angstroms) X	Y	Z
1	8	0	1.384885	-1.397330	-1.340366
2	8	0	-4.202774	-0.436074	-0.357512
3	8	0	2.687189	-1.595818	0.460522
4	7	0	3.497627	1.058557	-0.088837
5	6	0	1.135356	1.100491	0.764198
6	6	0	2.129512	0.613285	-0.311939
7	6	0	-0.290784	0.697564	0.507716
8	6	0	-1.080675	1.414360	-0.388049
9	6	0	-0.845860	-0.424014	1.114317
10	6	0	2.115902	-0.897758	-0.346841
11	6	0	-2.380732	1.032070	-0.670597
12	6	0	-2.146379	-0.822705	0.842671
13	6	0	-2.914905	-0.092159	-0.053207
14	1	0	1.220173	2.188870	0.788714
15	1	0	1.471371	0.720576	1.732142
16	1	0	1.796351	0.977192	-1.283142
17	1	0	-0.670757	2.292755	-0.873836
18	1	0	-0.250592	-1.002685	1.811939
19	1	0	3.798602	0.740217	0.826671
20	1	0	3.495974	2.071332	-0.049565
21	1	0	-2.988924	1.599389	-1.364048
22	1	0	-2.567588	-1.697549	1.324637
23	1	0	1.376751	-2.363686	-1.271670
24	1	0	-4.447755	-1.229350	0.129246

Tyrosine in acetonitrile

opt freq wb97xd scrf=(smd,solvent= acetonitrile) def2tzvp

Standard orientation:

Center Number	Atomic Number	Atomic Type	Coordinates (Angstroms) X	Y	Z
1	8	0	1.456090	-1.407487	-1.360040
2	8	0	-4.208657	-0.438391	-0.365852
3	8	0	2.695140	-1.579674	0.485440
4	7	0	3.484763	1.080979	-0.086221
5	6	0	1.121153	1.098731	0.769568

6	6	0	2.122025	0.620017	-0.306135
7	6	0	-0.303439	0.691517	0.507599
8	6	0	-1.089589	1.401130	-0.398103
9	6	0	-0.864557	-0.423064	1.121221
10	6	0	2.139540	-0.895365	-0.336251
11	6	0	-2.388691	1.018736	-0.683876
12	6	0	-2.164750	-0.822135	0.846408
13	6	0	-2.930944	-0.099709	-0.060047
14	1	0	1.200254	2.187791	0.799615
15	1	0	1.455569	0.718135	1.737891
16	1	0	1.785282	0.978062	-1.279522
17	1	0	-0.677380	2.275951	-0.889078
18	1	0	-0.276558	-0.995769	1.830083
19	1	0	3.812960	0.750891	0.815106
20	1	0	3.488612	2.093507	-0.045292
21	1	0	-2.992370	1.581910	-1.385016
22	1	0	-2.586251	-1.692625	1.337515
23	1	0	1.473997	-2.374448	-1.284326
24	1	0	-4.462530	-1.224983	0.127873

Phenylalanine in water

opt freq wb97xd scrf=(smd,solvent=water) def2tzvp

Standard orientation:

Center Number	Atomic Number	Atomic Type	Coordinates (Angstroms) X	Y	Z
1	8	0	-1.095854	-1.328389	-1.353225
2	8	0	-2.443841	-1.496492	0.416962
3	7	0	-3.023869	1.233001	0.018780
4	6	0	-0.648876	1.051692	0.815314
5	6	0	-1.701846	0.691137	-0.255885
6	6	0	0.738558	0.573663	0.486350
7	6	0	1.256160	-0.583011	1.061369
8	6	0	1.514433	1.261290	-0.444229
9	6	0	-1.806084	-0.813590	-0.352448
10	6	0	2.519686	-1.043247	0.715928
11	6	0	2.776337	0.805022	-0.792335
12	6	0	3.283139	-0.350792	-0.212688
13	1	0	-0.979668	0.640310	1.771746
14	1	0	-0.658642	2.140274	0.899644
15	1	0	-1.360150	1.069654	-1.218462
16	1	0	0.660663	-1.129977	1.783994
17	1	0	1.122002	2.163734	-0.899578

18	1	0	-2.937589	2.238068	0.116552
19	1	0	-3.338342	0.886181	0.919223
20	1	0	2.908813	-1.943833	1.174858
21	1	0	3.367459	1.353600	-1.515411
22	1	0	4.269348	-0.708297	-0.481654
23	1	0	-1.158294	-2.294655	-1.320527

Phenylalanine in acetonitrile

opt freq wb97xd scrf=(smd,solvent= acetonitrile) def2tzvp

Standard orientation:

Center Number	Atomic Number	Atomic Type	Coordinates (Angstroms) X	Y	Z
1	8	0	-1.167788	-1.328114	-1.383214
2	8	0	-2.430443	-1.490188	0.447281
3	7	0	-3.014408	1.242386	0.030817
4	6	0	-0.635567	1.052217	0.818776
5	6	0	-1.696470	0.694739	-0.247891
6	6	0	0.749973	0.570562	0.484042
7	6	0	1.277267	-0.574690	1.073385
8	6	0	1.517083	1.246431	-0.462873
9	6	0	-1.822246	-0.812862	-0.342204
10	6	0	2.539876	-1.036076	0.724839
11	6	0	2.777566	0.788739	-0.814653
12	6	0	3.293166	-0.356459	-0.221498
13	1	0	-0.962700	0.643074	1.777515
14	1	0	-0.640966	2.141099	0.904333
15	1	0	-1.358626	1.071174	-1.213819
16	1	0	0.692199	-1.111092	1.812537
17	1	0	1.119382	2.141588	-0.928463
18	1	0	-2.941195	2.248641	0.124739
19	1	0	-3.348273	0.888495	0.920860
20	1	0	2.936273	-1.927732	1.195567
21	1	0	3.360795	1.327936	-1.551543
22	1	0	4.278515	-0.715180	-0.493160
23	1	0	-1.252590	-2.293895	-1.348367

Thymine in water

opt freq wb97xd scrf=(smd,solvent=water) def2tzvp

Standard orientation:

Center Number	Atomic Number	Atomic Type	Coordinates (Angstroms) X	Y	Z
1	8	0	1.362403	1.886830	-0.000270
2	8	0	-2.855096	0.223988	0.000218
3	7	0	-0.723597	1.022750	-0.000345
4	7	0	-1.108240	-1.234654	0.000175
5	6	0	1.147267	-0.476785	0.000061
6	6	0	0.653681	0.885298	-0.000087
7	6	0	0.239543	-1.467454	0.000305
8	6	0	2.621956	-0.711438	0.000169
9	6	0	-1.649981	0.013571	-0.000428
10	1	0	0.520068	-2.511648	0.000629
11	1	0	-1.092143	1.964847	-0.000329
12	1	0	-1.752626	-2.010690	0.000589
13	1	0	3.088009	-0.262779	0.879900
14	1	0	3.088121	-0.262928	-0.879578
15	1	0	2.838174	-1.779178	0.000274

Thymine in acetonitrile

opt freq wb97xd scrf=(smd,solvent= acetonitrile) def2tzvp

Standard orientation:

Center Number	Atomic Number	Atomic Type	Coordinates (Angstroms) X	Y	Z
1	8	0	1.361773	1.888070	-0.000267
2	8	0	-2.857140	0.221102	0.000238
3	7	0	-0.725227	1.022440	-0.000323
4	7	0	-1.107601	-1.236020	0.000170
5	6	0	1.149327	-0.477601	0.000055
6	6	0	0.658365	0.892721	-0.000057
7	6	0	0.240919	-1.467186	0.000313
8	6	0	2.624450	-0.713522	0.000168
9	6	0	-1.659016	0.014795	-0.000529
10	1	0	0.522303	-2.511883	0.000675
11	1	0	-1.092659	1.964295	-0.000279
12	1	0	-1.747886	-2.014827	0.000615
13	1	0	3.092419	-0.264644	0.879147
14	1	0	3.092528	-0.264801	-0.878831
15	1	0	2.841758	-1.781693	0.000279

Adenine in water

opt freq wb97xd scrf=(smd,solvent=water) def2tzvp

Standard orientation:

Center Number	Atomic Number	Atomic Type	Coordinates (Angstroms) X	Y	Z
1	7	0	1.275645	1.309331	-0.000037
2	7	0	2.088118	-0.766197	-0.000127
3	7	0	-0.043023	-1.927040	-0.000005
4	7	0	-1.948721	-0.472545	0.000052
5	7	0	-1.799352	1.833921	-0.000097
6	6	0	0.190451	0.466070	-0.000006
7	6	0	0.716765	-0.816048	-0.000016
8	6	0	-1.199483	0.635681	-0.000002
9	6	0	2.365480	0.518672	0.000030
10	6	0	-1.335080	-1.662444	0.000156
11	1	0	1.273287	2.316539	-0.000088
12	1	0	3.359545	0.937095	0.000012
13	1	0	-2.001515	-2.518006	-0.000143
14	1	0	-2.803044	1.885631	0.000371
15	1	0	-1.265742	2.684860	0.000368

Adenine in acetonitrile

opt freq wb97xd scrf=(smd,solvent= acetonitrile) def2tzvp

Standard orientation:

Center Number	Atomic Number	Atomic Type	Coordinates (Angstroms) X	Y	Z
1	7	0	1.278921	1.307993	-0.000043
2	7	0	2.087820	-0.764944	-0.000155
3	7	0	-0.045401	-1.924826	-0.000014
4	7	0	-1.945216	-0.471954	0.000061
5	7	0	-1.805613	1.832706	-0.000075
6	6	0	0.192428	0.468556	-0.000005
7	6	0	0.717786	-0.817569	-0.000024
8	6	0	-1.199995	0.637089	0.000005
9	6	0	2.370311	0.517657	0.000039
10	6	0	-1.337540	-1.664349	0.000169
11	1	0	1.279142	2.315545	-0.000094
12	1	0	3.365000	0.935883	0.000020
13	1	0	-2.005401	-2.519915	-0.000151

14	1	0	-2.810203	1.878371	0.000344
15	1	0	-1.280053	2.688984	0.000356

Guanine in water

opt freq wb97xd scrf=(smd,solvent=water) def2tzvp

Standard orientation:

Center Number	Atomic Number	Atomic Type	Coordinates (Angstroms) X	Y	Z
1	8	0	0.087875	2.640650	-0.000105
2	7	0	-2.187056	0.517185	0.000214
3	7	0	1.452990	0.812277	-0.000124
4	7	0	0.732168	-1.437025	0.000029
5	7	0	-1.673810	-1.647922	0.000572
6	7	0	2.973985	-0.914835	-0.000106
7	6	0	-0.815110	0.433665	0.000045
8	6	0	-0.519463	-0.916386	-0.000029
9	6	0	0.201454	1.413152	0.000043
10	6	0	1.686014	-0.540072	-0.000099
11	6	0	-2.637548	-0.744509	-0.000422
12	1	0	-2.747565	1.354211	0.000394
13	1	0	2.244717	1.441947	-0.000212
14	1	0	-3.692516	-0.967612	-0.000413
15	1	0	3.192908	-1.894728	-0.000054
16	1	0	3.719427	-0.241872	-0.000200

Guanine in acetonitrile

opt freq wb97xd scrf=(smd,solvent= acetonitrile) def2tzvp

Standard orientation:

Center Number	Atomic Number	Atomic Type	Coordinates (Angstroms) X	Y	Z
1	8	0	0.088439	2.640962	-0.000125
2	7	0	-2.188134	0.513652	0.000263
3	7	0	1.454950	0.812244	-0.000154
4	7	0	0.734490	-1.434683	0.000055
5	7	0	-1.675077	-1.646381	0.000649
6	7	0	2.976902	-0.918858	-0.000121
7	6	0	-0.817350	0.433701	0.000053

8	6	0	-0.520186	-0.916826	0.000004
9	6	0	0.199717	1.424247	0.000030
10	6	0	1.688312	-0.539616	-0.000119
11	6	0	-2.642242	-0.748062	-0.000505
12	1	0	-2.748733	1.351130	0.000459
13	1	0	2.246669	1.441629	-0.000256
14	1	0	-3.697664	-0.971229	-0.000552
15	1	0	3.192907	-1.899893	-0.000045
16	1	0	3.727883	-0.251823	-0.000235

Cytosine in water

opt freq wb97xd scrf=(smd,solvent=water) def2tzvp

Standard orientation:

Center Number	Atomic Number	Atomic Type	Coordinates (Angstroms) X	Y	Z
1	8	0	-2.227815	-1.133715	0.000069
2	7	0	0.012690	-0.915888	0.000002
3	7	0	2.317481	-0.843837	0.000015
4	7	0	-1.377376	0.985626	0.000007
5	6	0	1.156978	-0.197587	-0.000003
6	6	0	1.029299	1.190257	-0.000056
7	6	0	-1.263329	-0.358288	0.000032
8	6	0	-0.250430	1.696781	-0.000196
9	1	0	0.057737	-1.926390	0.000022
10	1	0	1.902272	1.824223	-0.000010
11	1	0	-0.378691	2.774520	0.000097
12	1	0	3.176623	-0.321731	0.000227
13	1	0	2.359906	-1.849175	0.000278

Cytosine in acetonitrile

opt freq wb97xd scrf=(smd,solvent= acetonitrile) def2tzvp

Standard orientation:

Center Number	Atomic Number	Atomic Type	Coordinates (Angstroms) X	Y	Z
1	8	0	-2.226659	-1.135507	0.000073
2	7	0	0.015643	-0.915204	0.000000
3	7	0	2.321177	-0.842990	0.000012

4	7	0	-1.376141	0.988668	0.000003
5	6	0	1.157991	-0.196023	-0.000005
6	6	0	1.031035	1.191761	-0.000055
7	6	0	-1.275160	-0.365432	0.000043
8	6	0	-0.254757	1.697163	-0.000202
9	1	0	0.064580	-1.925383	0.000017
10	1	0	1.903185	1.827281	-0.000004
11	1	0	-0.383447	2.776507	0.000098
12	1	0	3.182475	-0.324294	0.000229
13	1	0	2.367073	-1.848190	0.000283

Ciprofloxacin in water

opt freq wb97xd scrf=(smd,solvent=water) def2tzvp

Standard orientation:

Center Number	Atomic Number	Atomic Type	Coordinates (Angstroms) X	Y	Z
1	9	0	-2.067396	-2.805551	-0.581416
2	8	0	2.880372	-2.594492	-0.326855
3	8	0	5.370707	-1.598407	0.153435
4	8	0	5.558318	0.600877	0.411931
5	7	0	1.551358	1.236991	0.209181
6	7	0	-3.061518	-0.258409	-0.151543
7	7	0	-5.713886	0.549911	0.544286
8	6	0	1.100845	2.601759	0.392594
9	6	0	1.861330	3.717297	-0.241698
10	6	0	0.510238	3.335214	-0.768449
11	6	0	0.620977	0.213874	0.028750
12	6	0	2.847528	0.949172	0.273981
13	6	0	1.080996	-1.092479	-0.143389
14	6	0	-0.747209	0.492952	0.047611
15	6	0	-1.692538	-0.505847	-0.126022
16	6	0	-3.850926	-0.988031	0.852347
17	6	0	-3.471440	1.141063	-0.214943
18	6	0	3.383565	-0.306998	0.122730
19	6	0	2.508918	-1.430148	-0.133174
20	6	0	-5.330239	-0.857027	0.567348
21	6	0	-4.956697	1.238386	-0.494668
22	6	0	0.132117	-2.104511	-0.332700
23	6	0	-1.195164	-1.811503	-0.332684
24	6	0	4.848478	-0.374383	0.239923
25	1	0	0.666006	2.771565	1.368734
26	1	0	2.717159	3.465259	-0.853161

27	1	0	1.961063	4.626427	0.334120
28	1	0	-0.336998	3.975277	-0.564960
29	1	0	0.480576	2.811981	-1.714839
30	1	0	3.502875	1.787332	0.460769
31	1	0	-1.074888	1.504948	0.215507
32	1	0	-3.632863	-0.581063	1.849395
33	1	0	-3.575921	-2.038380	0.850774
34	1	0	-2.925453	1.636567	-1.017610
35	1	0	-3.244578	1.662201	0.726037
36	1	0	-5.551426	-1.358733	-0.385755
37	1	0	-5.883981	-1.372535	1.352199
38	1	0	-5.238990	2.291009	-0.514242
39	1	0	-5.154780	0.818991	-1.491565
40	1	0	0.455356	-3.123088	-0.499434
41	1	0	-6.698344	0.611769	0.318921
42	1	0	6.330221	-1.515587	0.250960

Ciprofloxacin in acetonitrile

opt freq wb97xd scrf=(smd,solvent= acetonitrile) def2tzvp

Standard orientation:

Center Number	Atomic Number	Atomic Type	Coordinates (Angstroms) X	Y	Z
1	9	0	-2.093299	-2.780995	-0.559856
2	8	0	2.862352	-2.601743	-0.362742
3	8	0	5.369649	-1.621929	0.200653
4	8	0	5.573584	0.585559	0.317814
5	7	0	1.564784	1.235066	0.213169
6	7	0	-3.060872	-0.233966	-0.094725
7	7	0	-5.734258	0.529493	0.463563
8	6	0	1.127776	2.599240	0.417287
9	6	0	1.889796	3.716074	-0.215589
10	6	0	0.531438	3.347792	-0.733047
11	6	0	0.625254	0.218059	0.043662
12	6	0	2.862254	0.933135	0.264476
13	6	0	1.073691	-1.090609	-0.140813
14	6	0	-0.742058	0.503698	0.083056
15	6	0	-1.695345	-0.489598	-0.087138
16	6	0	-3.868958	-0.962614	0.889721
17	6	0	-3.480182	1.157164	-0.207928
18	6	0	3.389595	-0.322132	0.106767
19	6	0	2.505858	-1.447946	-0.151455
20	6	0	-5.339341	-0.869393	0.535942

21	6	0	-4.957009	1.222901	-0.552311
22	6	0	0.117852	-2.095212	-0.326148
23	6	0	-1.209013	-1.795695	-0.310151
24	6	0	4.858682	-0.389921	0.212693
25	1	0	0.703353	2.764563	1.399416
26	1	0	2.738752	3.464070	-0.836815
27	1	0	2.000532	4.620767	0.365814
28	1	0	-0.308571	3.994660	-0.519612
29	1	0	0.490741	2.830258	-1.682527
30	1	0	3.528371	1.764416	0.445637
31	1	0	-1.065718	1.515465	0.262695
32	1	0	-3.706773	-0.528096	1.886808
33	1	0	-3.565767	-2.005096	0.921352
34	1	0	-2.906351	1.638536	-1.000029
35	1	0	-3.300501	1.703195	0.730069
36	1	0	-5.506010	-1.396222	-0.416442
37	1	0	-5.923545	-1.371285	1.308605
38	1	0	-5.264475	2.268822	-0.592866
39	1	0	-5.102126	0.785776	-1.552546
40	1	0	0.442935	-3.111730	-0.504405
41	1	0	-6.719395	0.596418	0.244172
42	1	0	6.330230	-1.524460	0.285375

Astaxanthin in water

opt freq wb97xd scrf=(smd,solvent=water) def2tzvp

Standard orientation:

Center Number	Atomic Number	Atomic Type	Coordinates (Angstroms) X	Y	Z
1	8	0	15.078343	2.071556	-0.024677
2	8	0	-15.078583	-2.071085	-0.025043
3	8	0	12.970443	2.740196	-1.471153
4	8	0	-12.970613	-2.739801	-1.471370
5	6	0	12.681058	-0.732911	0.766208
6	6	0	-12.681021	0.733029	0.766365
7	6	0	13.722802	0.364738	1.005766
8	6	0	-13.722858	-0.364572	1.005744
9	6	0	11.545737	-0.232185	-0.130782
10	6	0	-11.545765	0.232379	-0.130743
11	6	0	14.119892	1.065405	-0.271369
12	6	0	-14.119996	-1.065009	-0.271512
13	6	0	12.140833	-1.154799	2.138092
14	6	0	13.331911	-1.955363	0.094417

15	6	0	-12.140717	1.154606	2.138313
16	6	0	-13.331780	1.955676	0.094829
17	6	0	11.663182	0.845809	-0.945976
18	6	0	-11.663279	-0.845501	-0.946073
19	6	0	12.894377	1.640985	-0.935092
20	6	0	-12.894505	-1.640628	-0.935236
21	6	0	10.330400	-1.047597	-0.074259
22	6	0	-10.330360	1.047686	-0.074118
23	6	0	10.614019	1.330146	-1.906297
24	6	0	-10.614151	-1.329809	-1.906445
25	6	0	9.078097	-0.575078	-0.028673
26	6	0	-9.078107	0.575044	-0.028523
27	6	0	7.875036	-1.390934	0.069487
28	6	0	-7.874970	1.390784	0.069680
29	6	0	8.030473	-2.881062	0.115000
30	6	0	-8.030275	2.880923	0.115336
31	6	0	6.687188	-0.749443	0.101864
32	6	0	-6.687169	0.749195	0.101923
33	6	0	5.372899	-1.341102	0.169643
34	6	0	-5.372835	1.340766	0.169580
35	6	0	4.252330	-0.596631	0.178240
36	6	0	-4.252301	0.596242	0.178186
37	6	0	2.892376	-1.107934	0.220901
38	6	0	-2.892330	1.107513	0.220676
39	6	0	2.697919	-2.594384	0.260536
40	6	0	-2.697843	2.593968	0.260009
41	6	0	1.880251	-0.210797	0.214641
42	6	0	-1.880227	0.210347	0.214589
43	6	0	0.466567	-0.486211	0.226733
44	6	0	-0.466538	0.485736	0.226557
45	1	0	14.605335	-0.070936	1.478232
46	1	0	13.312867	1.111140	1.692723
47	1	0	-13.312993	-1.111111	1.692592
48	1	0	-14.605361	0.071105	1.478263
49	1	0	14.526908	0.336354	-0.985744
50	1	0	-14.526895	-0.335801	-0.985789
51	1	0	12.977923	-1.422493	2.786749
52	1	0	11.592145	-0.337583	2.610763
53	1	0	11.479988	-2.019503	2.071868
54	1	0	14.134433	-2.334441	0.730474
55	1	0	12.606623	-2.758707	-0.043612
56	1	0	13.752271	-1.711187	-0.882062
57	1	0	-12.977761	1.422238	2.787055
58	1	0	-11.592074	0.337255	2.610802
59	1	0	-11.479804	2.019269	2.072229
60	1	0	-14.134282	2.334671	0.730960
61	1	0	-12.606434	2.759000	-0.043012

62	1	0	-13.752147	1.711746	-0.881710
63	1	0	10.490423	-2.116586	0.015615
64	1	0	-10.490291	2.116683	0.015816
65	1	0	10.014530	2.136880	-1.477825
66	1	0	9.934876	0.530324	-2.194432
67	1	0	11.088110	1.727116	-2.804496
68	1	0	-9.934926	-0.530015	-2.194462
69	1	0	-10.014747	-2.136661	-1.478076
70	1	0	-11.088276	-1.726615	-2.804701
71	1	0	14.861240	2.800914	-0.621625
72	1	0	-14.861250	-2.800556	-0.621774
73	1	0	8.914822	0.497442	-0.044562
74	1	0	-8.914936	-0.497492	-0.044507
75	1	0	7.077285	-3.396679	0.201277
76	1	0	8.651506	-3.175902	0.964925
77	1	0	8.531613	-3.243716	-0.786087
78	1	0	-8.651552	3.175707	0.965100
79	1	0	-8.531086	3.243760	-0.785861
80	1	0	-7.077064	3.396427	0.202016
81	1	0	6.707917	0.337170	0.063419
82	1	0	-6.707983	-0.337414	0.063415
83	1	0	5.293480	-2.421292	0.205914
84	1	0	-5.293345	2.420956	0.205694
85	1	0	4.352897	0.485704	0.141263
86	1	0	-4.352914	-0.486094	0.141358
87	1	0	2.148443	0.842875	0.189440
88	1	0	-2.148450	-0.843323	0.189661
89	1	0	3.149352	-3.063635	-0.617168
90	1	0	1.649353	-2.879321	0.293008
91	1	0	3.190002	-3.021540	1.137878
92	1	0	-3.149474	3.063086	-0.617662
93	1	0	-3.189719	3.021268	1.137400
94	1	0	-1.649270	2.878904	0.292199
95	1	0	0.133122	-1.519246	0.228142
96	1	0	-0.133072	1.518764	0.227558

Astaxanthin in acetonitrile

opt freq wb97xd scrf=(smd,solvent= acetonitrile) def2tzvp

Standard orientation:

Center Number	Atomic Number	Atomic Type	Coordinates (Angstroms) X	Y	Z
1	8	0	15.052747	2.092075	0.073344

2	8	0	-15.052686	-2.092237	0.073195
3	8	0	13.070181	2.551326	-1.597719
4	8	0	-13.069987	-2.551510	-1.597720
5	6	0	12.596267	-0.605229	1.034280
6	6	0	-12.596359	0.605181	1.034175
7	6	0	13.607100	0.533607	1.213606
8	6	0	-13.607185	-0.533664	1.213475
9	6	0	11.547562	-0.251583	-0.025243
10	6	0	-11.547545	0.251489	-0.025229
11	6	0	14.121944	1.057355	-0.107932
12	6	0	-14.121901	-1.057500	-0.108078
13	6	0	11.928102	-0.841013	2.394904
14	6	0	13.314700	-1.897574	0.605648
15	6	0	-11.928320	0.841052	2.394847
16	6	0	-13.314771	1.897490	0.605416
17	6	0	11.730682	0.713297	-0.958649
18	6	0	-11.730570	-0.713446	-0.958600
19	6	0	12.955717	1.526921	-0.944855
20	6	0	-12.955598	-1.527079	-0.944886
21	6	0	10.335354	-1.076350	0.024645
22	6	0	-10.335386	1.076327	0.024704
23	6	0	10.766264	1.055133	-2.059184
24	6	0	-10.766096	-1.055317	-2.059070
25	6	0	9.082032	-0.614821	-0.063819
26	6	0	-9.082025	0.614851	-0.063475
27	6	0	7.873629	-1.425531	0.028656
28	6	0	-7.873659	1.425623	0.028976
29	6	0	8.026686	-2.906882	0.202189
30	6	0	-8.026748	2.907007	0.202234
31	6	0	6.686138	-0.787382	-0.050294
32	6	0	-6.686153	0.787492	-0.049898
33	6	0	5.367333	-1.372437	-0.000536
34	6	0	-5.367352	1.372577	-0.000360
35	6	0	4.249974	-0.625554	-0.061832
36	6	0	-4.249986	0.625694	-0.061566
37	6	0	2.885645	-1.127717	-0.031376
38	6	0	-2.885656	1.127880	-0.031396
39	6	0	2.680394	-2.611644	0.046570
40	6	0	-2.680388	2.611834	0.046098
41	6	0	1.879627	-0.224488	-0.075263
42	6	0	-1.879644	0.224636	-0.075135
43	6	0	0.463391	-0.489027	-0.069086
44	6	0	-0.463405	0.489161	-0.069250
45	1	0	14.441930	0.184338	1.825027
46	1	0	13.130856	1.362617	1.746244
47	1	0	-13.130967	-1.362633	1.746201
48	1	0	-14.442074	-0.184380	1.824805

49	1	0	14.585418	0.236640	-0.674697
50	1	0	-14.585356	-0.236833	-0.674929
51	1	0	12.699279	-0.997121	3.153104
52	1	0	11.328617	0.022059	2.692997
53	1	0	11.282935	-1.720528	2.389160
54	1	0	14.061751	-2.166690	1.355511
55	1	0	12.612155	-2.728429	0.522149
56	1	0	13.817326	-1.785608	-0.356111
57	1	0	-12.699566	0.997236	3.152963
58	1	0	-11.328886	-0.022013	2.693062
59	1	0	-11.283129	1.720548	2.389095
60	1	0	-14.061895	2.166633	1.355196
61	1	0	-12.612232	2.728352	0.521937
62	1	0	-13.817308	1.785463	-0.356383
63	1	0	10.495422	-2.130852	0.224291
64	1	0	-10.495549	2.130854	0.224139
65	1	0	10.128000	1.899976	-1.788292
66	1	0	10.118113	0.214968	-2.301771
67	1	0	11.315490	1.347628	-2.954847
68	1	0	-10.117848	-0.215215	-2.301599
69	1	0	-10.127938	-1.900234	-1.788150
70	1	0	-11.315277	-1.347744	-2.954784
71	1	0	14.835602	2.758194	-0.594028
72	1	0	-14.835532	-2.758364	-0.594161
73	1	0	8.920955	0.450369	-0.196299
74	1	0	-8.920875	-0.450354	-0.195752
75	1	0	7.070664	-3.421178	0.264994
76	1	0	8.587461	-3.130175	1.113931
77	1	0	8.588472	-3.336697	-0.631310
78	1	0	-8.588302	3.130459	1.113454
79	1	0	-8.587739	3.336806	-0.631813
80	1	0	-7.070737	3.421220	0.265865
81	1	0	6.710847	0.293471	-0.168760
82	1	0	-6.710849	-0.293380	-0.168186
83	1	0	5.282147	-2.449204	0.087763
84	1	0	-5.282172	2.449370	0.087624
85	1	0	4.356941	0.453701	-0.145574
86	1	0	-4.356951	-0.453584	-0.145010
87	1	0	2.155881	0.826550	-0.121289
88	1	0	-2.155920	-0.826412	-0.120788
89	1	0	3.154679	-3.109470	-0.803228
90	1	0	1.629238	-2.889571	0.055435
91	1	0	3.142340	-3.015951	0.951090
92	1	0	-3.154778	3.109432	-0.803773
93	1	0	-3.142214	3.016393	0.950567
94	1	0	-1.629233	2.889766	0.054763
95	1	0	0.123015	-1.519928	-0.065203

96	1	0	-0.123015	1.520058	-0.065898

$C_4H_6Cl_2$ and OOH in water

opt freq wb97xd scrf=(smd,solvent= water) def2tzvp

Standard orientation:

Center Number	Atomic Number	Atomic Type	Coordinates (Angstroms) X	Y	Z
1	6	0	1.512742	0.568785	-0.362375
2	1	0	1.321593	0.444858	-1.425553
3	6	0	2.420366	1.751070	-0.097717
4	1	0	2.629874	1.849817	0.967936
5	1	0	3.359298	1.644271	-0.639966
6	1	0	1.921994	2.660184	-0.440081
7	17	0	2.422298	-0.977837	0.099469
8	6	0	0.261876	0.616235	0.406519
9	1	0	0.297416	0.814875	1.471178
10	6	0	-1.028562	0.303950	-0.199609
11	1	0	-1.082834	0.472361	-1.272266
12	17	0	-1.349962	-1.543552	-0.048609
13	8	0	-2.020485	0.991184	0.482495
14	8	0	-3.226965	0.926706	-0.254657
15	1	0	-3.695982	0.193886	0.170532

$C_4H_6Cl_2$ and OOH in water

opt freq wb97xd scrf=(smd,solvent= water) def2tzvp

Standard orientation:

Center Number	Atomic Number	Atomic Type	Coordinates (Angstroms) X	Y	Z
1	6	0	0.082946	1.532608	-0.241022
2	6	0	-0.825704	1.603193	-1.445291
3	1	0	-1.506034	0.753052	-1.483622
4	1	0	-1.401850	2.528467	-1.434231
5	1	0	-0.207674	1.589015	-2.343398
6	17	0	-2.757257	-1.778076	-0.148955
7	6	0	0.856843	0.260411	-0.133200
8	1	0	0.310200	-0.673623	-0.192752
9	6	0	2.170464	0.281270	-0.006130

10	1	0	2.758017	1.188187	0.047153
11	17	0	3.127630	-1.159446	0.071917
12	1	0	0.751216	2.391436	-0.205070
13	8	0	-0.700074	1.741418	0.999206
14	8	0	-1.619971	0.857299	1.205578
15	1	0	-2.147152	-0.693273	0.237154

$C_4H_6Cl_2$ and OOH in acetonitrile

opt freq wb97xd scrf=(smd,solvent= acetonitrile) def2tzvp

Standard orientation:

Center Number	Atomic Number	Atomic Type	Coordinates (Angstroms) X	Y	Z
1	6	0	1.507672	0.549171	-0.368434
2	1	0	1.309828	0.384830	-1.425226
3	6	0	2.402858	1.753211	-0.158146
4	1	0	2.617669	1.898617	0.901330
5	1	0	3.340202	1.634550	-0.701373
6	1	0	1.892345	2.641924	-0.535461
7	17	0	2.438117	-0.965925	0.144761
8	6	0	0.260563	0.612529	0.407776
9	1	0	0.306251	0.814704	1.471488
10	6	0	-1.037026	0.321875	-0.191693
11	1	0	-1.101105	0.509422	-1.261028
12	17	0	-1.351672	-1.545274	-0.095437
13	8	0	-2.017343	0.975442	0.514963
14	8	0	-3.230018	0.930224	-0.212175
15	1	0	-3.660274	0.140287	0.152430

$C_4H_6Cl_2$ and OOH in acetonitrile

opt freq wb97xd scrf=(smd,solvent= acetonitrile) def2tzvp

Standard orientation:

Center Number	Atomic Number	Atomic Type	Coordinates (Angstroms) X	Y	Z
1	6	0	0.079988	-1.540075	0.240577
2	6	0	-0.831315	-1.626643	1.442573
3	1	0	-1.521745	-0.784493	1.486473

4	1	0	-1.398005	-2.558096	1.427108
5	1	0	-0.216328	-1.610179	2.343082
6	17	0	-2.740327	1.791301	0.152078
7	6	0	0.851924	-0.264778	0.147774
8	1	0	0.309706	0.669599	0.239649
9	6	0	2.163526	-0.281218	-0.003194
10	1	0	2.750709	-1.186540	-0.085842
11	17	0	3.118479	1.161326	-0.068653
12	1	0	0.752039	-2.396401	0.200058
13	8	0	-0.700463	-1.741855	-1.003787
14	8	0	-1.614859	-0.852435	-1.211889
15	1	0	-2.167117	0.702048	-0.269728

$C_4H_6Cl_2$ and ciprofloxacin in water

opt freq wb97xd scrf=(smd,solvent= water) def2tzvp

Standard orientation:

Center Number	Atomic Number	Atomic Type	Coordinates (Angstroms) X	Y	Z
1	9	0	-2.006081	-1.529369	-2.529015
2	8	0	2.919090	-1.752944	-2.065747
3	8	0	5.384133	-1.727306	-0.899866
4	8	0	5.510539	-1.003535	1.197779
5	7	0	1.480755	-0.675094	1.607702
6	7	0	-3.082981	-1.004193	-0.025583
7	7	0	-5.788901	-1.319963	0.843494
8	6	0	0.996160	-0.219859	2.894532
9	6	0	1.703903	0.894474	3.590563
10	6	0	0.351209	1.125650	2.986939
11	6	0	0.581307	-0.951223	0.580026
12	6	0	2.786054	-0.817735	1.401328
13	6	0	1.080437	-1.317739	-0.670124
14	6	0	-0.794651	-0.878877	0.807528
15	6	0	-1.710834	-1.108976	-0.208144
16	6	0	-3.883219	-2.175650	-0.411080
17	6	0	-3.539361	-0.468087	1.251969
18	6	0	3.358394	-1.205215	0.214023
19	6	0	2.515595	-1.452606	-0.934459
20	6	0	-5.353676	-1.823882	-0.454270
21	6	0	-5.014230	-0.132434	1.183119
22	6	0	0.162680	-1.530398	-1.705785
23	6	0	-1.170810	-1.403415	-1.480869
24	6	0	4.826672	-1.292959	0.231693

25	1	0	0.584995	-1.018140	3.498235
26	1	0	2.549132	1.350022	3.092046
27	1	0	1.793709	0.815326	4.664728
28	1	0	-0.510657	1.215616	3.633437
29	1	0	0.314100	1.711100	2.077192
30	1	0	3.416065	-0.604003	2.252483
31	1	0	-1.150285	-0.649796	1.797637
32	1	0	-3.719394	-2.980029	0.319099
33	1	0	-3.571451	-2.538441	-1.385088
34	1	0	-2.982146	0.438702	1.483597
35	1	0	-3.363899	-1.188098	2.063943
36	1	0	-5.516089	-1.087901	-1.255000
37	1	0	-5.920242	-2.720802	-0.705097
38	1	0	-5.332542	0.244641	2.154987
39	1	0	-5.155729	0.673513	0.448125
40	1	0	0.518288	-1.762750	-2.700249
41	1	0	-6.764546	-1.060021	0.778402
42	1	0	6.343078	-1.745225	-0.768647
43	6	0	-1.151067	3.852484	-0.915210
44	6	0	-0.010236	3.502303	-1.482173
45	6	0	0.658281	2.174790	-1.410151
46	1	0	0.042863	1.442642	-0.892697
47	6	0	1.082727	1.668076	-2.770392
48	1	0	1.687948	2.411533	-3.291070
49	1	0	-1.587936	4.834002	-1.029737
50	1	0	1.651259	0.743410	-2.685921
51	1	0	0.186477	1.470306	-3.362559
52	17	0	2.134932	2.353691	-0.343051
53	1	0	0.488004	4.260971	-2.077657
54	17	0	-2.109667	2.807512	0.085401

$C_4H_6Cl_2$ and ciprofloxacin in acetonitrile

opt freq wb97xd scrf=(smd,solvent= acetonitrile) def2tzvp

Standard orientation:

Center Number	Atomic Number	Atomic Type	Coordinates (Angstroms) X	Y	Z
1	9	0	-2.037591	0.210762	-2.762219
2	8	0	2.917280	0.061948	-2.626082
3	8	0	5.451920	-0.506029	-1.740666
4	8	0	5.646675	-1.341578	0.306307
5	7	0	1.637993	-1.522511	0.921257
6	7	0	-2.989917	-0.955724	-0.433652

7	7	0	-5.658566	-1.699775	0.173249
8	6	0	1.212467	-2.077200	2.188119
9	6	0	1.917148	-1.662053	3.437808
10	6	0	0.519205	-1.190053	3.173200
11	6	0	0.695560	-1.132872	-0.028895
12	6	0	2.936930	-1.439158	0.635152
13	6	0	1.138424	-0.599595	-1.239537
14	6	0	-0.669481	-1.285029	0.224756
15	6	0	-1.628794	-0.873190	-0.689003
16	6	0	-3.813851	-1.681294	-1.405696
17	6	0	-3.394129	-1.241930	0.936387
18	6	0	3.460358	-0.944776	-0.531356
19	6	0	2.568167	-0.446368	-1.565219
20	6	0	-5.280774	-1.364876	-1.191744
21	6	0	-4.867377	-0.928508	1.118858
22	6	0	0.177089	-0.170605	-2.161637
23	6	0	-1.148759	-0.290171	-1.882188
24	6	0	4.933154	-0.954613	-0.596576
25	1	0	0.873441	-3.102374	2.109313
26	1	0	2.708267	-0.928963	3.352669
27	1	0	2.079355	-2.427392	4.184041
28	1	0	-0.299641	-1.621565	3.732550
29	1	0	0.394635	-0.152005	2.893645
30	1	0	3.606878	-1.791427	1.406390
31	1	0	-0.986116	-1.742206	1.146854
32	1	0	-3.653940	-2.762180	-1.283513
33	1	0	-3.524809	-1.409325	-2.416541
34	1	0	-2.810045	-0.620977	1.615599
35	1	0	-3.211865	-2.296430	1.191267
36	1	0	-5.447199	-0.299913	-1.417100
37	1	0	-5.877721	-1.956773	-1.887001
38	1	0	-5.160613	-1.189732	2.136674
39	1	0	-5.014071	0.155418	0.990616
40	1	0	0.498343	0.289366	-3.086600
41	1	0	-6.641760	-1.507644	0.313271
42	1	0	6.414773	-0.570742	-1.651354
43	6	0	-1.578801	3.677100	1.000782
44	6	0	-0.269790	3.694711	0.819742
45	6	0	0.556303	2.625057	0.194295
46	1	0	-0.017325	1.713886	0.046064
47	6	0	1.200516	3.072557	-1.100258
48	1	0	1.774888	3.988918	-0.954600
49	1	0	-2.121869	4.504047	1.435837
50	1	0	1.855753	2.298985	-1.499540
51	1	0	0.411972	3.269128	-1.830498
52	17	0	1.853481	2.147855	1.388508
53	1	0	0.252523	4.596159	1.124679

54	17	0	-2.606451	2.350698	0.555189

$C_{14}H_{21}Cl_7$ and OOH in water

opt freq wb97xd scrf=(smd,solvent= water) def2tzvp

Standard orientation:

Center Number	Atomic Number	Atomic Type	Coordinates (Angstroms) X	Y	Z
1	6	0	-2.598144	0.111134	-0.113185
2	1	0	-2.692265	-0.698938	-0.831995
3	6	0	-3.771045	0.114783	0.851160
4	1	0	-3.725270	0.999566	1.489120
5	1	0	-3.671644	-0.757359	1.502073
6	17	0	-2.637028	1.619240	-1.132967
7	6	0	-1.279268	0.014189	0.628625
8	1	0	-1.316616	-0.891671	1.240254
9	1	0	-1.189557	0.861981	1.313555
10	6	0	-0.027400	-0.011493	-0.226912
11	6	0	1.226006	-0.072991	0.625012
12	1	0	1.226563	-1.006314	1.194576
13	1	0	1.175043	0.744951	1.348624
14	6	0	2.542743	0.006720	-0.123764
15	1	0	2.575341	-0.729085	-0.923884
16	17	0	2.664398	1.608048	-0.985264
17	6	0	3.723095	-0.172572	0.811694
18	1	0	3.588686	-1.130323	1.321918
19	1	0	3.697164	0.607671	1.577362
20	6	0	5.099199	-0.134920	0.175674
21	6	0	6.195469	-0.281882	1.213188
22	1	0	6.087496	-1.246447	1.717175
23	1	0	6.037772	0.494383	1.967372
24	6	0	7.622096	-0.195695	0.706626
25	1	0	7.788905	-0.910291	-0.096113
26	17	0	7.905628	1.426001	-0.082874
27	6	0	8.638441	-0.391935	1.807674
28	1	0	8.499013	-1.383430	2.244116
29	1	0	9.654265	-0.326723	1.419323
30	1	0	8.511242	0.352977	2.594999
31	6	0	-5.143814	0.099832	0.202806
32	6	0	-6.240851	0.107759	1.215135
33	1	0	-6.293263	-0.748615	1.879369
34	6	0	-7.127144	1.068150	1.401152
35	1	0	-7.882844	1.029443	2.172420

36	17	0	-7.203494	2.525648	0.461640
37	17	0	-5.090597	-3.139482	0.710488
38	17	0	-0.074295	-1.439112	-1.358027
39	1	0	0.000315	0.850529	-0.889449
40	17	0	5.244001	-1.455611	-1.072208
41	1	0	5.233121	0.782933	-0.391845
42	1	0	-5.247987	0.930387	-0.496728
43	8	0	-5.194582	-1.093531	-0.613447
44	8	0	-6.406710	-1.074495	-1.317959
45	1	0	-6.147863	-1.508339	-2.143694

$C_{14}H_{21}Cl_7$ and OOH in acetonitrile

opt freq wb97xd scrf=(smd,solvent= acetonitrile) def2tzvp

Standard orientation:

Center Number	Atomic Number	Atomic Type	Coordinates (Angstroms) X	Y	Z
1	6	0	2.565281	-0.449087	-0.130355
2	1	0	2.678890	-0.190443	-1.180836
3	6	0	3.747512	0.050237	0.682425
4	1	0	3.666003	-0.305918	1.711844
5	1	0	3.684800	1.140844	0.722211
6	17	0	2.533365	-2.269434	-0.120393
7	6	0	1.261769	0.102766	0.416353
8	1	0	1.344349	1.193422	0.409649
9	1	0	1.153764	-0.205815	1.460006
10	6	0	-0.006981	-0.307655	-0.306571
11	6	0	-1.236226	0.278356	0.362457
12	1	0	-1.197052	1.368760	0.291186
13	1	0	-1.185301	0.023962	1.424761
14	6	0	-2.577609	-0.168197	-0.189267
15	1	0	-2.624482	-0.011264	-1.264609
16	17	0	-2.757022	-1.970809	0.006976
17	6	0	-3.719772	0.548860	0.506101
18	1	0	-3.549537	1.622039	0.381291
19	1	0	-3.674198	0.338471	1.578481
20	6	0	-5.123632	0.210753	0.040360
21	6	0	-6.166132	0.964655	0.844423
22	1	0	-6.016061	2.039668	0.708067
23	1	0	-5.981965	0.747437	1.900671
24	6	0	-7.620519	0.672667	0.527630
25	1	0	-7.820699	0.816626	-0.531868
26	17	0	-7.979257	-1.095696	0.807552

27	6	0	-8.564534	1.507012	1.363531
28	1	0	-8.382954	2.563509	1.152703
29	1	0	-9.604531	1.283991	1.126334
30	1	0	-8.399613	1.334931	2.428743
31	6	0	5.115858	-0.352505	0.171561
32	6	0	6.220936	0.145768	1.043484
33	1	0	6.065065	1.082010	1.568284
34	6	0	7.373313	-0.465650	1.256827
35	1	0	8.132501	-0.068071	1.915914
36	17	0	7.835328	-1.967221	0.529398
37	17	0	5.251989	3.978799	0.527077
38	17	0	0.061079	0.223720	-2.047774
39	1	0	-0.081375	-1.391158	-0.362545
40	17	0	-5.302815	0.598447	-1.731585
41	1	0	-5.299072	-0.861152	0.096972
42	1	0	5.182351	-1.430026	0.030274
43	8	0	5.304766	0.118587	-1.219929
44	8	0	5.331756	1.404870	-1.346485
45	1	0	5.276006	2.829933	-0.081220

$C_4H_7Cl_3$ and OOH in water

opt freq wb97xd scrf=(smd,solvent= water) def2tzvp

Standard orientation:

Center Number	Atomic Number	Atomic Type	Coordinates (Angstroms) X	Y	Z
1	6	0	1.247581	-0.349637	0.309482
2	1	0	1.154275	-1.349024	-0.120077
3	6	0	2.141255	0.487247	-0.579700
4	1	0	2.334048	1.471204	-0.161763
5	1	0	1.725070	0.592066	-1.579279
6	6	0	-0.154000	0.221826	0.420821
7	6	0	-1.100159	-0.629415	1.247164
8	17	0	-1.434454	-2.194453	0.427037
9	1	0	-0.674027	-0.864280	2.219798
10	1	0	-2.060325	-0.137367	1.368281
11	17	0	-0.114109	1.843371	1.236684
12	1	0	1.683224	-0.453003	1.305339
13	17	0	3.751938	-0.293592	-0.776232
14	1	0	-0.579777	0.411660	-0.562392
15	8	0	-3.326397	1.138407	-0.948430
16	8	0	-2.936617	0.458018	-1.990498

17	1	0	-3.743820	0.136677	-2.432397

$C_4H_7Cl_3$ and OOH in acetonitrile

opt freq wb97xd scrf=(smd,solvent= acetonitrile) def2tzvp

Standard orientation:

Center Number	Atomic Number	Atomic Type	Coordinates (Angstroms) X	Y	Z
1	6	0	1.231523	0.464106	-0.173442
2	1	0	1.230423	1.211303	0.622976
3	6	0	1.997795	-0.754367	0.295041
4	1	0	2.090913	-1.508687	-0.481686
5	1	0	1.543407	-1.198025	1.178447
6	6	0	-0.217737	0.148798	-0.497147
7	6	0	-1.041408	1.367488	-0.872630
8	17	0	-1.269221	2.446595	0.548749
9	1	0	-0.552372	1.957119	-1.645132
10	1	0	-2.036963	1.079475	-1.196871
11	17	0	-0.316767	-0.973119	-1.922366
12	1	0	1.713029	0.909429	-1.046648
13	17	0	3.680319	-0.308143	0.758838
14	1	0	-0.697232	-0.391711	0.317642
15	8	0	-2.389309	-1.875772	1.543724
16	8	0	-3.428072	-1.519900	0.835532
17	1	0	-4.176823	-2.060339	1.157526

$C_4H_7Cl_3$ and ciprofloxacin in water

opt freq wb97xd scrf=(smd,solvent= water) def2tzvp

Standard orientation:

Center Number	Atomic Number	Atomic Type	Coordinates (Angstroms) X	Y	Z
1	9	0	1.970880	0.087914	2.884655
2	8	0	-2.967745	-0.248301	2.581900
3	8	0	-5.407423	-1.127031	1.703082
4	8	0	-5.544192	-1.660227	-0.450328
5	7	0	-1.507175	-1.720842	-0.942691
6	7	0	3.050306	-1.013640	0.569958

7	7	0	5.742099	-1.847248	0.070455
8	6	0	-1.018642	-2.161406	-2.233345
9	6	0	-1.715165	-1.698240	-3.469652
10	6	0	-0.354700	-1.167645	-3.131964
11	6	0	-0.613213	-1.310247	0.043442
12	6	0	-2.813850	-1.702922	-0.699323
13	6	0	-1.116336	-0.820941	1.249768
14	6	0	0.762417	-1.405801	-0.170292
15	6	0	1.677332	-0.974247	0.778817
16	6	0	3.843724	-1.711342	1.592951
17	6	0	3.497858	-1.387919	-0.768044
18	6	0	-3.389811	-1.272642	0.470975
19	6	0	-2.553486	-0.742731	1.524610
20	6	0	5.318623	-1.434567	1.403619
21	6	0	4.976958	-1.108120	-0.926554
22	6	0	-0.200227	-0.368801	2.206819
23	6	0	1.135966	-0.429728	1.965319
24	6	0	-4.857098	-1.367007	0.512116
25	1	0	-0.622719	-3.168200	-2.215904
26	1	0	-2.548986	-1.018105	-3.355983
27	1	0	-1.814595	-2.417834	-4.269956
28	1	0	0.503695	-1.509154	-3.693498
29	1	0	-0.298975	-0.147086	-2.775530
30	1	0	-3.440919	-2.063141	-1.502043
31	1	0	1.117989	-1.834913	-1.091086
32	1	0	3.660121	-2.791729	1.516614
33	1	0	3.543150	-1.389304	2.584307
34	1	0	2.948015	-0.806222	-1.506401
35	1	0	3.303267	-2.452277	-0.961978
36	1	0	5.501421	-0.364663	1.579305
37	1	0	5.878176	-1.995386	2.152428
38	1	0	5.284733	-1.417690	-1.925379
39	1	0	5.139604	-0.023349	-0.847084
40	1	0	-0.557720	0.059504	3.133098
41	1	0	6.721584	-1.619346	-0.041473
42	1	0	-6.365542	-1.230417	1.612151
43	6	0	0.754233	2.972312	-2.431013
44	6	0	0.153623	3.439087	-1.125849
45	6	0	-0.574993	2.376135	-0.326745
46	1	0	0.038914	1.487935	-0.182554
47	6	0	-1.101601	2.870162	1.007583
48	1	0	-1.682578	3.782151	0.890601
49	1	0	1.190026	3.808450	-2.969816
50	1	0	-1.697113	2.108247	1.501879
51	17	0	-2.029538	1.801163	-1.247863
52	1	0	0.942212	3.855371	-0.496173
53	17	0	2.099096	1.794404	-2.173856

54	17	0	0.254948	3.253250	2.123123
55	1	0	0.030914	2.469733	-3.068622
56	1	0	-0.540615	4.252539	-1.353405

$C_4H_7Cl_3$ and ciprofloxacin in acetonitrile

opt freq wb97xd scrf=(smd,solvent= acetonitrile) def2tzvp

Standard orientation:

Center Number	Atomic Number	Atomic Type	Coordinates (Angstroms) X	Y	Z
1	9	0	1.985134	0.671588	2.711296
2	8	0	-2.955705	0.382138	2.422106
3	8	0	-5.416177	-0.678515	1.796167
4	8	0	-5.543833	-1.824467	-0.101243
5	7	0	-1.508624	-1.969195	-0.589181
6	7	0	3.054150	-0.970214	0.745478
7	7	0	5.757524	-1.784873	0.451459
8	6	0	-1.023825	-2.763689	-1.696344
9	6	0	-1.721083	-2.679336	-3.013990
10	6	0	-0.360253	-2.073105	-2.845742
11	6	0	-0.610086	-1.338635	0.269512
12	6	0	-2.817386	-1.894847	-0.346403
13	6	0	-1.109576	-0.561859	1.315011
14	6	0	0.766180	-1.496899	0.094116
15	6	0	1.681988	-0.863279	0.923112
16	6	0	3.843322	-1.417987	1.898790
17	6	0	3.522769	-1.581939	-0.491224
18	6	0	-3.392692	-1.176399	0.669027
19	6	0	-2.553195	-0.384100	1.551590
20	6	0	5.314011	-1.131290	1.673464
21	6	0	4.999166	-1.292831	-0.688157
22	6	0	-0.193752	0.093986	2.144139
23	6	0	1.144006	-0.042003	1.938223
24	6	0	-4.863265	-1.260092	0.730463
25	1	0	-0.627917	-3.723585	-1.389847
26	1	0	-2.556546	-1.997403	-3.102683
27	1	0	-1.819532	-3.600174	-3.571847
28	1	0	0.496559	-2.561667	-3.289174
29	1	0	-0.305584	-0.993266	-2.797577
30	1	0	-3.447545	-2.452781	-1.024244
31	1	0	1.125294	-2.139374	-0.692135
32	1	0	3.700244	-2.498935	2.039452
33	1	0	3.508390	-0.913529	2.799565

34	1	0	2.965246	-1.165425	-1.329635
35	1	0	3.361650	-2.670082	-0.479285
36	1	0	5.457659	-0.040242	1.635125
37	1	0	5.884206	-1.518850	2.518929
38	1	0	5.339447	-1.796022	-1.594250
39	1	0	5.127421	-0.208658	-0.832836
40	1	0	-0.557827	0.738453	2.932889
41	1	0	6.743313	-1.609852	0.307304
42	1	0	-6.372759	-0.815928	1.722924
43	6	0	0.897703	2.676559	-2.836189
44	6	0	0.161776	3.305222	-1.676171
45	6	0	-0.576288	2.341778	-0.766632
46	1	0	0.076459	1.545923	-0.410122
47	6	0	-1.268210	3.014589	0.404834
48	1	0	-1.909191	3.828604	0.072338
49	1	0	1.356862	3.443167	-3.454072
50	1	0	-1.845142	2.299824	0.985333
51	17	0	-1.887194	1.494086	-1.692555
52	1	0	0.869045	3.869567	-1.065030
53	17	0	2.247308	1.607787	-2.288633
54	17	0	-0.064887	3.724521	1.537741
55	1	0	0.252745	2.056210	-3.453449
56	1	0	-0.551059	4.024685	-2.089845

$C_{14}H_{22}Cl_8$ and OOH in water

opt freq wb97xd scrf=(smd,solvent= water) def2tzvp

Standard orientation:

Center Number	Atomic Number	Atomic Type	Coordinates (Angstroms) X	Y	Z
1	6	0	-6.505584	0.556464	-0.138545
2	1	0	-6.453601	0.981716	0.861132
3	6	0	-7.453994	1.359999	-0.998076
4	1	0	-7.635190	0.877361	-1.955435
5	1	0	-7.027382	2.347440	-1.161864
6	6	0	-5.131334	0.521681	-0.785639
7	6	0	-4.044641	-0.199580	-0.010673
8	17	0	-3.814193	0.591841	1.613082
9	1	0	-4.350099	-1.215534	0.228145
10	17	0	-9.050020	1.618829	-0.223828
11	1	0	-4.821696	1.556267	-0.954954
12	8	0	-0.576940	3.114577	-0.927542
13	8	0	-0.587322	2.851343	0.349504

14	1	0	-0.812795	1.906112	0.438171
15	6	0	-2.739435	-0.197472	-0.783391
16	1	0	-2.935991	-0.659505	-1.754810
17	6	0	-1.560209	-0.893608	-0.131337
18	1	0	-1.376415	-0.484103	0.861057
19	6	0	-0.308111	-0.762612	-0.978109
20	1	0	-0.198610	0.305476	-1.186270
21	6	0	0.979859	-1.239276	-0.304616
22	1	0	0.807292	-1.435803	0.751234
23	6	0	2.096166	-0.224681	-0.472180
24	6	0	3.434615	-0.556463	0.159770
25	1	0	3.810519	-1.508673	-0.209943
26	6	0	4.449152	0.538101	-0.116584
27	1	0	4.108395	1.487894	0.297811
28	6	0	5.884737	0.247472	0.316984
29	1	0	6.031425	-0.818816	0.483964
30	6	0	6.891715	0.754643	-0.703281
31	1	0	6.766304	1.830503	-0.843535
32	1	0	6.645017	0.266653	-1.649223
33	6	0	8.324370	0.429190	-0.342683
34	1	0	8.663875	0.964954	0.538864
35	1	0	8.468145	-0.639135	-0.195670
36	17	0	9.436815	0.911968	-1.676282
37	17	0	6.231700	1.004226	1.939181
38	1	0	4.454788	0.656957	-1.203554
39	1	0	1.730139	0.718266	-0.055094
40	17	0	3.220539	-0.828850	1.946620
41	1	0	2.268934	-0.057550	-1.539114
42	17	0	1.500167	-2.850615	-0.971397
43	1	0	-0.449087	-1.251491	-1.942321
44	17	0	-1.953768	-2.643025	0.176258
45	1	0	-2.441586	0.837861	-0.973112
46	1	0	-5.207886	0.041458	-1.764784
47	17	0	-7.137959	-1.124305	0.095412

$C_{14}H_{22}Cl_8$ and OOH in acetonitrile

opt freq wb97xd scrf=(smd,solvent= acetonitrile) def2tzvp

Standard orientation:

Center Number	Atomic Number	Atomic Type	Coordinates (Angstroms) X	Y	Z
1	6	0	-6.360919	-0.094766	-0.533682
2	1	0	-6.513401	0.427236	0.408662

3	6	0	-7.277382	0.470960	-1.594683
4	1	0	-7.259052	-0.126559	-2.503382
5	1	0	-6.966895	1.489246	-1.821065
6	6	0	-4.914938	0.012554	-0.990842
7	6	0	-3.867405	-0.470857	-0.007467
8	17	0	-3.956314	0.522978	1.530172
9	1	0	-4.086819	-1.478786	0.335477
10	17	0	-8.985784	0.571291	-1.060153
11	1	0	-4.722725	1.060699	-1.235948
12	8	0	-4.112340	3.584747	-0.619397
13	8	0	-3.353298	3.497974	0.440938
14	1	0	-3.479400	2.585778	0.789594
15	6	0	-2.471923	-0.387903	-0.593406
16	1	0	-2.480126	-0.967365	-1.521225
17	6	0	-1.330295	-0.872628	0.285602
18	1	0	-1.317074	-0.325389	1.226359
19	6	0	0.014122	-0.740285	-0.426629
20	1	0	-0.088671	0.058160	-1.167133
21	6	0	1.192118	-0.331541	0.440645
22	1	0	0.929563	0.549302	1.024176
23	6	0	2.425800	-0.066574	-0.403836
24	6	0	3.670357	0.400160	0.330603
25	1	0	3.926117	-0.305674	1.117784
26	6	0	4.852901	0.590457	-0.621606
27	1	0	4.979614	1.643059	-0.876938
28	6	0	6.168985	0.005587	-0.140259
29	1	0	6.028041	-1.036710	0.145162
30	6	0	7.253506	0.121189	-1.199233
31	1	0	7.460533	1.174280	-1.400946
32	1	0	6.849506	-0.318695	-2.114390
33	6	0	8.525499	-0.606857	-0.820732
34	1	0	9.011414	-0.167751	0.046140
35	1	0	8.343733	-1.663987	-0.638432
36	17	0	9.729618	-0.523242	-2.159817
37	17	0	6.716199	0.814171	1.398678
38	1	0	4.622483	0.067681	-1.553633
39	1	0	2.156341	0.676730	-1.159497
40	17	0	3.307968	1.944593	1.225301
41	1	0	2.692872	-0.984133	-0.936209
42	17	0	1.568789	-1.595252	1.695019
43	1	0	0.243119	-1.651485	-0.981708
44	17	0	-1.642231	-2.597962	0.774920
45	1	0	-2.256748	0.649000	-0.866278
46	1	0	-4.783711	-0.564619	-1.910081
47	17	0	-6.788021	-1.820622	-0.187983
